# Strategies for chiral separation: from racemate to enantiomer

**DOI:** 10.1039/d3sc01630g

**Published:** 2023-09-27

**Authors:** Jingchen Sui, Na Wang, Jingkang Wang, Xin Huang, Ting Wang, Lina Zhou, Hongxun Hao

**Affiliations:** a National Engineering Research Center of Industrial Crystallization Technology, School of Chemical Engineering and Technology, Tianjin University Tianjin 300072 P. R. China wangna224@tju.edu.cn hongxunhao@tju.edu.cn +86-22-2740-5754; b Collaborative Innovation Center of Chemical Science and Engineering Tianjin 300072 P. R. China; c School of Chemical Engineering and Technology, Hainan University Haikou 570228 China

## Abstract

Chiral separation has become a crucial topic for effectively utilizing superfluous racemates synthesized by chemical means and satisfying the growing requirements for producing enantiopure chiral compounds. However, the remarkably close physical and chemical properties of enantiomers present significant obstacles, making it necessary to develop novel enantioseparation methods. This review comprehensively summaries the latest developments in the main enantioseparation methods, including preparative-scale chromatography, enantioselective liquid–liquid extraction, crystallization-based methods for chiral separation, deracemization process coupling racemization and crystallization, porous material method and membrane resolution method, focusing on significant cases involving crystallization, deracemization and membranes. Notably, potential trends and future directions are suggested based on the state-of-art “coupling” strategy, which may greatly reinvigorate the existing individual methods and facilitate the emergence of cross-cutting ideas among researchers from different enantioseparation domains.

## Abstract 2

Research progress and potential trends about six critical chiral resolution methods are summarized. Notable performance improvements based on the state-of-the-art “coupling” strategies are highlighted.

## Graphical abstract

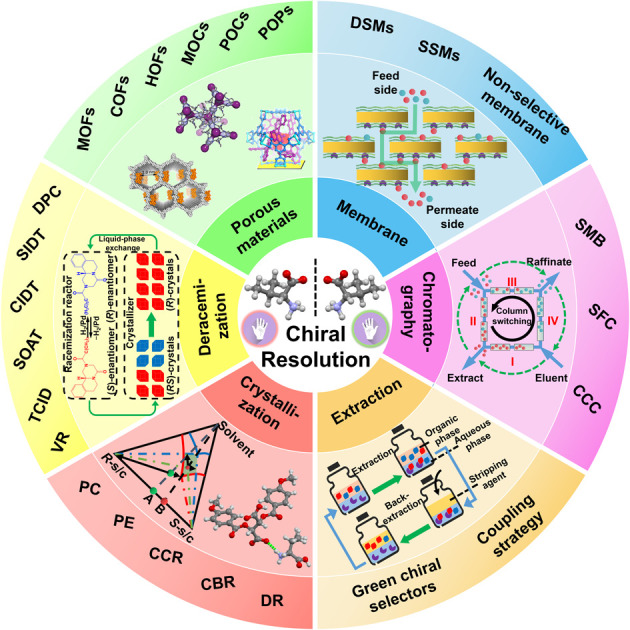

## 1. Introduction

Chirality means that an object cannot coincide with its mirror image. Generally, enantiomers, possessing at least one chiral center, chiral axis, or chiral plane, are stereoisomers that cannot be superimposed with their mirror image. Generally, they are designated as d (dextro) or l (levo), *R* (rectus) or *S* (sinister), (+) or (−), and P (plus) or M (minus).^1^ As one of the basic attributes of nature and the universe, chirality is widely used in various fields, such as biology, medicine, life science, and materials science.^2–6^ Based on a comprehensive estimate, the global market value of chiral compounds is expected to exceed 96.8 billion U.S. dollars by 2024.^7^

Chiral drugs, which account for more than 72% of the chiral market and provide more precise care for humans, are the leader in the chiral compound market and this trend continues to increase.^7,8^ In 2015, among the 33 new molecular entity drugs and 13 biologics license applications approved by the U.S. Food and Drug Administration (FDA), 94.4% were chiral drugs with a clearly defined absolute configuration.^8,9^ Moreover, chiral pesticides, which are essential for increasing food production, are also expected to soar by about 14% in the next two years.^7,10^ Therefore, the development of chiral resolution techniques in industry and academia is significant.

As the cornerstone of new developments, prevailing resolution methods and their performance are summarized in Table 1. The application of preparative-scale chromatography (PsC) appears to be less widespread compared to analytical methods due to the use of a large amount of solvent and limited chiral stationary phases (CSP) under high-pressure conditions.^14^ However, three expedient operational modes of PsC have been presented to enhance the productivity and reduce the solvent consumption (Section 2).^7,15,42–45^ By comparison, enantioselective liquid–liquid extraction (ELLE) can offer a low solvent consumption process and overcome the utilization of CSPs at the expense of enantioselectivity.^21,46–48^ However, commonly used chiral selectors (CSs) suffer from high volatility, flammability and biotoxicity, and are generally specialized for resolving amino acids.^11,16–21^ Therefore, amino acid- and cyclodextrin-based chiral ionic liquids (CILs) and deep eutectic systems (DES) are emerging alternatives for more eco-friendly and generic ELLE processes.^49,50^ Moreover, the above-mentioned dilemma can be ingeniously tackled by combining ELLE with other enantioseparation methods, such as crystallization, deracemization, and membrane processes, leveraging their complementary advantages (Section 3).^51–53^

**Table 1:** Present techniques for chiral resolution

Method	Yield, ee, productivity	Cost	Strategies that can be coupled	Green chemistry	Application	Recent publication volume	Ref.
PsC	<50%	Costly	ELLE; deracemization	Low	Narrow	Medium	11–15
	High						
	Medium						
ELLE	<50%	Medium	CBR; KR; membrane; deracemization	Medium	Narrow	Numerous	11 and 16–21
	Low						
	High						
PC	<50%	Cheap	External field; membrane; deracemization; cocrystal	High	Broad	Medium	14 and 22
	High						
	High						
KR	<50%	Costly	Membrane; deracemization	Medium	Medium	Numerous	23 and 24
	High						
	Medium						
CCR	<50%	Medium	External field; PC; deracemization; ELLE	Low	Broad	Less	25 and 26
	High						
	High						
CBR	<50%	Medium	PE; PC; deracemization	High	Medium	Numerous	27 and 28
	High						
	High						
DR	<50%	Medium	—	Medium	Narrow	Less	29–32
	Medium						
	High						
PMs	<50%	Costly	PsC; membrane	High	Broad	Numerous	33 and 34
	Medium						
	Low						
Membrane	<50% medium	Cheap	ELLE; PC; KR; PsC	Medium	Medium	Numerous	35 and 36
	Medium						
Deracemization	≥50%	Cheap	As noted above	High	Medium	Numerous	37–41
	High						
	High						

Currently, a broad variety of substrates can be successfully resolved *via* classical chemical resolution (CCR, Section 4.4.2) with high yield, high enantiomeric excess (ee), and acceptable cost. Nevertheless, CCR cannot be regarded as an eco-friendly process and fails to exhibit enantiospecific recognition toward enantiomers,^54^ reducing its popularity in recent publications. Fortuitously, in the past decade, cocrystal-based resolution (CBR, Section 4.4.3) and Dutch resolution (DR, Section 4.4.4) have shown great potential to address the aforementioned issues in CCR by replacing strong acid–base interactions with non-covalent interactions (hydrogen bonding, halogen bonding, π–π interactions, van der Waals interaction, *etc.*), which makes the resolution and liberation of enantiomers greener and milder and extends the substrate scope of CCR.^55,56^ Hence, designing enantioselective cocrystals based on the Cambridge Structural Database (CSD), supramolecular synthons, molecular complementarity, thermodynamics and steric hindrance is important.^57–59^ Alternatively, preferential crystallization (PC, Section 4.2) can resolve racemic conglomerates with merits such as continuous operation, high productivity and ee value, as well as low cost.^14^ However, the limited proportion of conglomerates (<10%) and control of the crystallization kinetics are still challenges to be addressed. Recently, population balance models (PBMs),^60^ multi-vessel setups,^61–64^ and tailor-made chiral additives^65,66^ have been proposed to estimate the best stop time of the process and mitigate the contamination of undesirable enantiomers in the product. Besides, converting racemates into conglomerates through cocrystal formation also serves as an effective tool to expand the applicability of PC (Section 4.1, Section 4.4.3).^27^ As a highly beneficial process, preferential enrichment (PE, Section 4.3) can counterintuitively enantioenrich the target enantiomer in the liquid phase, rather than crystals. However, although some prerequisites have been proposed, its poorly understood mechanism and relatively limited range of eligible racemates restrict its application.^67^

In recent years, various chiral porous materials (PMs, Section 6), including MOFs, COFs, HOFs, MOCs and POCs,^14,68–71^ have emerged as novel CSPs and resolving agents, which is attributed to their excellent host–guest interactions, such as hydrogen bonding, halogen bonding, π–π interactions, hydrophobic interactions, van der Waals forces, and steric hindrance.^72–74^ However, these highly functionable framework structures may not be ideal in terms of price and efficiency. Furthermore, many PMs are loaded on a membrane matrix to create chiral solid membranes through methods such as phase conversion, *in situ* growth, coating, and non-covalent interactions, due to the advantages of low solvent and energy consumption, high process continuity, and uniformly distributed chiral recognition sites.^7,14,68^ Specifically, a large variety of membranes related to graphene oxide, macrocycles, amino acids, MOFs, COFs, and even target enantiomers has been developed for remarkable resolution outcomes (Section 7).^75–79^ Additionally, their mechanism can be analyzed using density functional theory (DFT), molecular docking and molecular dynamics (MD) simulations.^80,81^ Nevertheless, their large-scale industrial application is hindered by the cumbersome regeneration and trade-off between permselectivity and permeability of membranes.

Compared to other methods, deracemization can achieve 100% yield by adding a racemizing agent to totally transform a racemate to the desired enantiomer. Theoretically, it can be coupled with various enantioseparation methods such as PsC,^82^ ELLE,^53^ and kinetic resolution (KR).^83^ However, crystallization possesses the highest compatibility with deracemization (Section 5). This is because the size-dependent solubility difference between two enantiomers can be naturally and easily induced by simple physical processes during crystallization, such as boiling,^84^ agitation,^85^ temperature cycling,^86^ homogenization^87^ and grinding,^88^ thereby leading to a concentration difference between enantiomers for initializing racemization. Among them, temperature cycling-induced deracemization (TCID) and attrition-enhanced deracemization (VR) are the most widely used autocatalysis-crystallization techniques, which can be coupled with CCR-, CBR- and (reverse) PC-related processes^37,84,89,90^ to expand the substrate scope from racemic conglomerates (Con) to racemic compounds (Rac) and racemic solid solutions (Ss).^40,85,91^

To date, although several reviews have been published with regard to enantioseparation, few have systematically summarized all these strategies. Therefore, this review highlights the research progress on crystallization-based resolution and deracemization (Sections 4 and 5) and the membrane resolution method (Section 7). Importantly, the necessity of “coupling” strategies (Table 1) will be discussed, which may impart new vitality and overcome the limitations for individual resolution processes.

## 2. Chromatography

Chromatographic techniques, which rely on high-efficient chiral stationary phases (CSPs) or chiral selectors (CSs), have been widely employed to resolve almost all chiral compounds.^1^ However, PsC techniques have more stringent requirements with respect to the loadability and stereoselectivity of CSP/CS compared to analytical techniques. Currently, the available preparative-scale CSPs are dominated by macrocycles due to the limited design principles and compatibility.^92,93^ Some potential CSPs are introduced in Sections 6 and 7, while this section focuses on the operating mode.

**Fig. 1:**
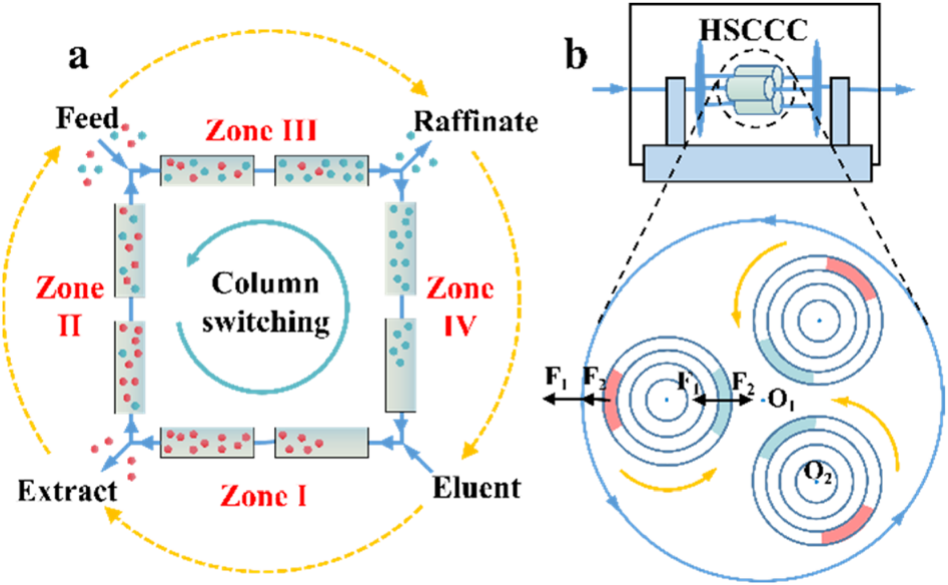
(a) Schematic representation of simulated moving bed chromatography. The orange dashed arrows represent the switching direction of the inlets and outlets in the VARICOL mode. (b) Schematic representation of high-speed countercurrent chromatography. The solid orange and blue arrows represent the rotation and revolution directions, respectively. *F*_1_ and *F*_2_ are centrifugal forces caused by revolution (*O*_1_ axis) and rotation (*O*_2_ axis), respectively. Red and blue circular are decanted and mixed “stationary phase + mobile phase”, respectively.

One significant preparative-scale high-performance liquid chromatography (HPLC) is simulated moving bed (SMB) chromatography. A typical SMB involves two separation zones (II and III) and two regeneration zones (I and IV) in a continuous process (Fig. 1a). After feeding the racemates, the strongly and weakly retained enantiomers are carried in opposite directions by the column and the mobile phase, respectively, and can be eluted and enriched in the extract phase and raffinate phase with the regeneration of the liquid (zone I) and solid phase (zone IV). The number of columns connected in series and the intervals of the column bed are adjustable and designable to obtain a reasonable peak shape and scaling ability. Additionally, a VARICOL process was proposed, in which the inlets and outlets are asynchronously switched along the direction of the fluid phase (Fig. 1a).^94^ This variant can adjust the number of columns in each zone, leading to a more rational distribution of CSPs and enhanced enantioselectivity. In this mode, successful resolutions of chiral drugs such as mitotane, guaifenesin and aminoglutethimide with chiral purities of 97%, 99% and over 99%, respectively, have been reported.^95,96^ ModiCon and PowerFeed are two additional variants for SMB, where the feed concentration and flow rate are manipulated to achieve better concentration distribution within the column, thereby generating more targeted extract and raffinate concentrations.^97,98^ Recently, a ModiCon and VariCol coupling process proved to double the maximum yield for guaifenesin enantioseparation because of the volume-enhanced I and II zones.^99^ Breveglieri *et al.* further integrated racemization kinetics with SMB technology, achieving a simultaneous reduction in the mass transfer resistance and 100% yield.^82,100^

Supercritical fluid chromatography (SFC) has the characteristics of both high-performance liquid chromatography (HPLC) and gas chromatography (GC) and often uses low-viscosity, high-diffusion supercritical CO_2_ or N_2_O as the mobile phase, which is beneficial to reduce the solvent consumption by 60–70% and operating cost by 70–80%.^43,101^ Additionally, the lower pressure drop in SFC has resulted in the development of new processes such as multi-column series, stacked injections and SFC-SMB coupling with adjustable parameters including the number of columns in series,^102^ the sequence of columns,^103,104^ chromatographic conditions and elution order.^105^ As a PsC, SFC can industrially resolve at least 95% of racemates and has emerged as a strong competitor to LC due to its excellent solvent compatibility, enantioselectivity and low residence time.^43,100^ Recently, Firooz *et al.* addressed the issue of SFC lacking polar CSPs by utilizing two newly modified cyclofructan columns, which exhibited remarkable selectivity for multiple polar and basic compounds.^106^ Akchich and colleagues reported 13 successful serial coupling processes by adopting 6 polysaccharide-based CSP columns. Enantiomers of dihydropyridine derivatives achieved baseline separation within 7 min with separation factors of 2.97, 1.83, and 3.54 on the tandem column system (OJ-H/AD-H).^107^ Michaels *et al.* pioneered a high-throughput SFC technique by integrating analytical and preparative-scale SFC. They characterized the separation effects of 50 racemates on 15 chromatographic columns, and 60–70% of the racemate exhibited preparation and screening performance on commercially available CSPs (Chiralpak IG, Whelk-O1, Chiralpak IA and Chiralpak IB).^108^ DaSilva *et al.* further discussed the cost and convenience benefits of high-speed SFC modes. Surprisingly, the Chiralpak ID column with 20 μm particles (about $4000) could achieve comparable outcomes to that with 5 μm particles (about $15000).^109^ By comparison, HPLC failed to realize this cost-saving potential. However, the recovery and operability of SFC are not as industrially favorable as LC because of the fluctuated solubility of compounds, making it a top priority for this “green technique” to ensure supercritical stablity.^15,110^

Compared to SMB and SFC, high-speed countercurrent chromatography (HSCCC) is a unique liquid–liquid partition chromatography without the participation of solid CSPs but chiral selectors (CSs). The propulsion force HSCCC is the partition coefficient difference of substances in multi-phase solvents. The stationary phase and mobile phase are alternatively decanted and mixed in spiral tubes when the orientations of centrifugal forces caused by high-speed rotation and revolution are the same and opposite, respectively (Fig. 1b). This imparts HSCCC with large single-stage load capacity, low cost, convenient scalability, fast separation speed and ease of coupling with online separation technology.^111–113^ At present, l-proline, proteins and β-cyclodextrin (CD) and its derivatives have become dominant chiral selectors in HSCCC.^114,115^ For example, Han *et al.* successfully separated racemic mandelic acid and its four derivatives in an average of 4–7 h by employing Cu_2_(ii)-β-CD as the chiral selector.^116^ Hydroxypropyl-β-cyclodextrin (HP-β-CD) was further applied to separate ketoconazole, ibuprofen and naringenin within 12.5 h, 7.3 h and 6.5 h, respectively.^117–119^ Also, *N-n*-dodecyl-l-hydroxyproline successfully resolved enantiomers such as phenylalanine, valine and isoleucine racemates on an HSCCC device within 2 h.^120^ Additionally, other variants such as high-performance centrifugal partition chromatography (HPCPC) and spiral tube CCC have attracted significant attention.^121–124^ Consequently, it is necessary to develop highly selective CSs, novel solvent systems and high-efficiency variants for the industrialization of CCC.^114,125,126^

However, despite their effectively increased separation performance and reduced solvent usage compared to HPLC, these modes still have some crucial aspects to consider, for instance, the stable control of supercritical fluids and the rational configuration of the column bed. In addition, preparative-scale CSPs are primarily subjected to polysaccharide and its derivatives. Therefore, it is indispensable to develop novel modes that can remain stable under high-pressure PsC conditions.

## 3. Enantioselective liquid–liquid extraction

ELLE is a technique that utilizes the enantioselective recognition between chiral selectors (CSs) and enantiomers in at least one liquid phase. According to the mutual solubility of the involved liquid phases, it can be classified into three cases, including biphasic recognition chiral extraction (BRCE, consisting of aqueous layer and organic layer),^127^ aqueous two-phase biphasic extraction (ATPE, immiscible water-rich phase formed by two water-soluble solutes exceeding a certain concentration),^128^ and synergistic extraction (involving two or more extractants in the traditional LLE process).^129^ However, the commonly used ELLE CSs, including tartaric acid and its derivatives,^130–132^ cyclodextrin,^133–135^ BINOL-based hosts,^47,136,137^ SPINOL-based hosts,^138^ VANOL-based hosts,^139^ and metal complexes,^128,140–143^ typically encounter challenges of high pollution, low selectivity, and need for additional solvents. Thus, to address these limitations, “tailor-made” chiral ionic liquids (CILs)^144^ or deep eutectic systems (DESs),^49,50^ composed of eutectic mixtures of H-bond donors and acceptors with remarkable structural diversity, non-volatility and thermal stability, have been innovatively designed as both CSs and phase formers. For example, Wang *et al.* described a greener and more efficient ELLE process by selecting hydroxypropyl-β-cyclodextrin (HP-β-CD) and (+)-diisopropyl l-tartrate as chiral selectors for hydrophilic and hydrophobic DESs, respectively.^145^ Consequently, this ELLE system exhibited a maximum enantioselectivity of 31.6% for threonine enantiomers. Similarly, Ma *et al.* successfully resolved tryptophan enantiomers with a chiral purity of 38.46% by optimizing the formulation of DESs.^146^ Consequently, CILs and DESs are potential candidates for designing more eco-friendly and cost-effective ELLE processes.

**Fig. 2:**
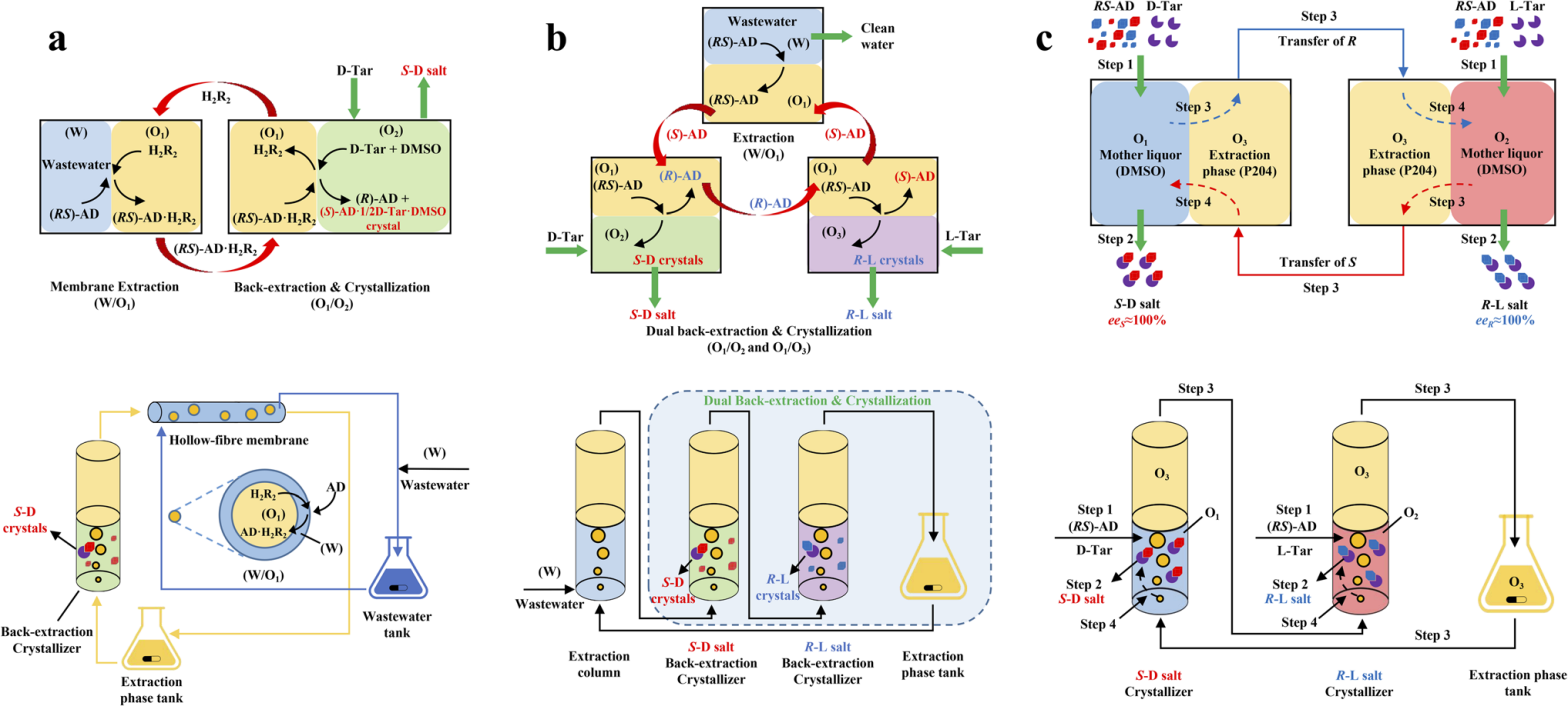
Mechanisms and schematic diagrams of (a) extraction-crystallization-membrane coupling method,^51^ (b) multiple-phase extraction and *in situ* coupling of crystallization,^52^ and (c) pairwise crystallization-circulating extraction coupling method for mother liquor *in situ* reuse.^147^

More importantly, it seems that coupling ELLE with other resolution methods is a promising strategy for enhancing the enantioseparation performance. For example, Zeng *et al.* innovatively combined hollow fiber membrane extraction with “*in situ* crystallization coupled back-extraction” to recover chiral drugs from pharmaceutical wastewater (Fig. 2a).^51^ Specifically, (*RS*)-amlodipine (AD) was extracted into the organic phase (O_1_) through oil-in-water emulsions in a hollow-fiber membrane, followed by further extraction into a heavier DMSO phase (O_2_) in the back extraction-crystallization device. Accordingly, the chiral resolving agent d-tartaric acid (d-Tar) could capture a high concentration of (*S*)-AD and precipitate (*S*)-AD·1/2d-Tar·DMSO (*S*-d salt) crystals with 90.7% ± 1.4% ee and 48.8% ± 2.4% yield. Also, the same group achieved the simultaneous preparation of both enantiomers by inserting an (*R*)-AD·1/2l-Tar·DMSO (*R*-l salt) crystallizer after the *S*-d salt crystallizer (Fig. 2b), thereby producing (*R*)- and (*S*)-enantiomers with optical purities of 94.3% and 92.2%, respectively.^52^ It is evident that these two coupling processes provide a high concentration of racemate for the occurrence of classical chemical resolution (CCR). In 2022, they further presented a pairwise crystallization-circulating extraction strategy (Fig. 2c), where the system achieved solid–liquid equilibrium to produce *S*-d and *R*-l salt crystals in their respective crystallizers (step 2). Taking dissolved (*R*)-AD as an example, it could be extracted from the d-Tar-enriched O_1_ phase to P204, where the liquid circulation took place (step 3), and then the l-Tar-enriched O_2_ phase to precipitate as the stable *R*-l salt (step 4). Consequently, crystals with ee values higher than 98% could be consecutively harvested after 15 cycles, and the overall yields reached nearly 100%.^147^ Hence, the integration of extraction, crystallization, membrane, and liquid-phase exchange processes shows promise for chiral resolution.

**Fig. 3:**
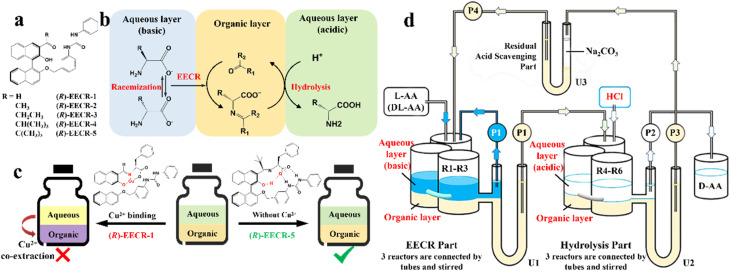
Cu^2+^ sequestration and EECR in continuous flow. (a) Carbonyl compounds studied for EECR in this work. (b) Enantioselective extraction coupled with racemization (EECR). (c) Schematic representation of the results using (*R*)-EECR-1 or (*R*)-EECR-5 as the extractant. (d) Schematic design of a continuous reaction system that carries out EECR and hydrolysis reactions repeatedly.^53^

In addition, although racemic amino acids (AAs) are the most suitable system for the ELLE technique, achieving complete deracemization toward the target enantiomer is still complex due to the prerequisite of –NH_2_ group derivatization before racemization. In 2021, Huang *et al.* presented a derivatization-free strategy *via* enantioselective extraction coupled with racemization (EECR) (Fig. 3).^53^ Specifically, the potential ketone CS ((*R*)-EECR-1–5) could enantiospecifically extract d-AA molecules from a basic aqueous solution into the organic phase to form a complex. Consequently, the excess l-AA remaining in the alkaline aqueous phase underwent continuous racemization catalyzed by the Cu^2+^/pyridoxal-5′-phosphate catalyst, further promoting the enrichment of d-AA molecules in the organic phase (Fig. 3b). Ultimately, the complex at the interface between the organic phase and acidic aqueous solution underwent hydrolysis and released the high-value-added d-AA products in the aqueous phase due to the ingenious design of the circulation and communicating vessels (Fig. 3d). Notably, among five CS candidates (Fig. 3a), (*R*)-EECR-1–4 inevitably incorporated Cu^2+^ into the complex because of the limited steric hindrance of their *R*-substituents, which may carry the racemizing agent into hydrolysis part, and consequently convert the desired d-AAs back to their antipodes (Fig. 3c, left). In contrast, (*R*)-EECR-5 possessing the largest substituents (tertiary butyl) hindered the formation of the hydrogen bond-assisted resonant and prevented the coextraction of Cu^2+^ ions from the alkaline aqueous solution to the organic layer (Fig. 3c, right), thereby hindering the racemization of the target enantiomer. After 40 h of continuous resolution, d-AAs with ee values of over 98% could be obtained from optically pure l- or dl-AAs.

The development of CILs and DESs has resulted in higher enantioselectivity and greener processes. However, the resolution performances achieved by the ELLE-coupled strategy are often several times greater than that achieved by ELLE alone. Consequently, ELLE is seldom employed independently in practical applications. Instead, it can serve as a vital enhancer for other enantioseparation methods.

## 4. Crystallization-based methods for chiral separation

As the most industrially favored technique, crystallization-based methods have witnessed steadfast progress since Pasteur hand-sorted enantiomers of sodium ammonium tartrate in 1848 (ref. 148) and utilized a resolving agent to separate enantiomers by forming diastereomeric salts in 1853.^149^ Specifically, the 1848 study underlies the foundation of spontaneous resolution free of external chiral sources, thereby contributing to methods such as PC, reverse PC, and PE, which rely on the control of crystallization kinetics. Alternatively, the 1853 study prompted the development of chemical resolution, such as KR, CCR, CBR, and DR, in which a chiral resolving agent is indispensable to discriminate enantiomers by forming diastereomers, reaction products, or complexes with distinct physical and chemical properties. However, the single crystallization strategy has notable limitations. For example, the substrate scopes of PC and PE are limited by the types of crystal packing of chiral molecules. CCR/CBR/DR is only suitable for batch operation and the desired yield is continuously lower than 50%. Recently, the crystallization-based enantioseparation performance has proved to be enhanced by integrating methods in the two above-mentioned branches such as PC-CBR, PC-cocrystal system, PE-cocrystal system, PC-CCR, (reverse) PC-external field, and PC variants. Moreover, crystallization showcases strong coupling capability with deracemization, membrane and ELLE, including KR-deracemization, CCR-deracemization, CBR-deracemization, CCR-ELLE, PC-deracemization, KR-membrane, and PC-membrane processes. Considering new developments related to (reverse) PC, PE, KR, CCR, CBR, and DR, there is potential for broader applications, particularly on an industrial scale. However, realizing this necessitates the development of continuous multidisciplinary crystallization designs based on the specific characteristics of each system. Hence, there are both opportunities and challenges ahead given the excellent separation performance and complexity of the process.

### 4.1 Racemic compound *vs.* conglomerate *vs.* solid solution

**Fig. 4:**
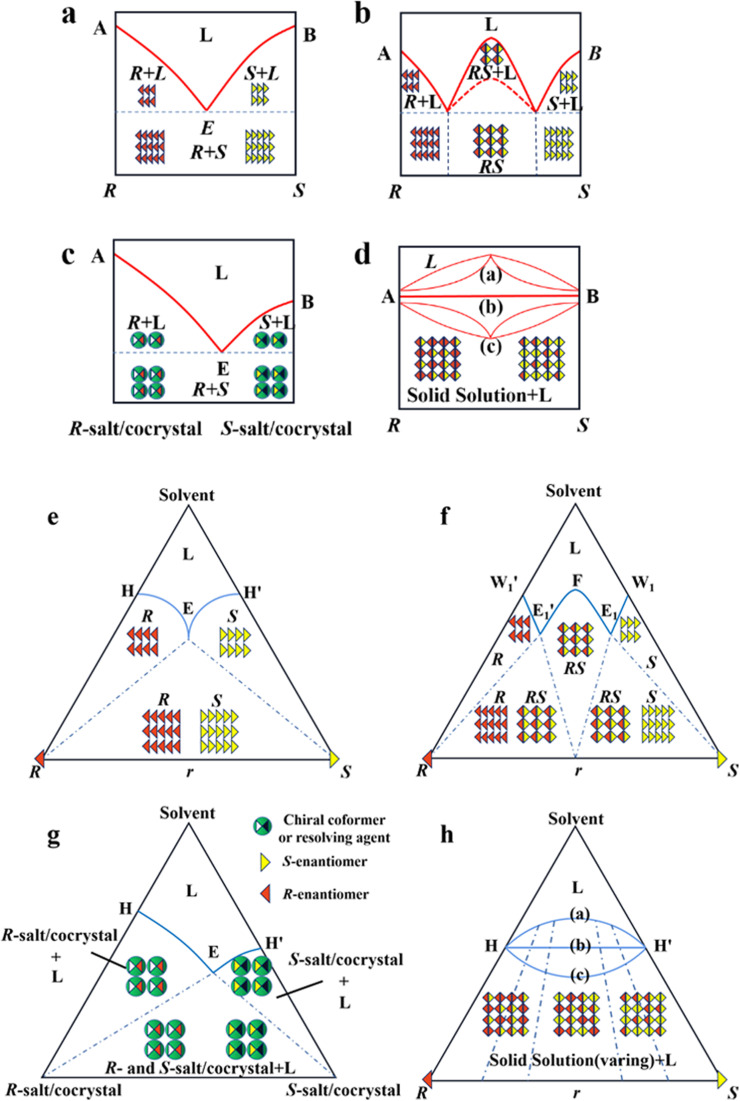
Scheme and phase diagrams of two- and three-component crystalline state of racemates. (a and e) conglomerate, (b and f) racemic compound, (c and g) diastereomeric pair and (d and h) solid solution, respectively.

As mentioned earlier, it is crucial to gain in-depth knowledge on the type of packing of racemates given that it serves as the basis for designing crystallization-based resolution processes. Left- and right-handed molecules possess identical functional groups and enantiomer connectivity. However, the crystalline arrangement of enantiomers (E: E^+^ or E^−^) relies on the relative strength of the interactions between chiral molecules and the steric hindrance in the crystal structure.^150^ When the intermolecular interactions between the antipodes exceed that of the single-handed molecules, chiral molecules constitute racemic compound (Rac) crystals, where E^+^ and E^−^ coexist in the crystal lattice (Fig. 4b and f, respectively). When the intermolecular interactions between heterochiral molecules are weaker than that of homochiral ones, E^+^ and E^−^ crystallize in their respective lattices and the crystals are physically mixed as a racemic conglomerate (Con) (Fig. 4a and e, respectively). When the intermolecular interactions between mirror images are similar, E^+^ and E^−^ are irregularly arranged in the crystal lattice and miscible with their counter-enantiomers, similar to a solid solution (Ss) (Fig. 4d and h, respectively). The reliable identification of racemates can be achieved by multifaceted methods including spectroscopy, thermodynamics, thermal analysis (Table 2). It can be seen that most Con crystals possess lower thermodynamic stability and share the same signal positions with a single enantiomer. This characteristic facilitates the ease of resolving Con. By comparison, most racemates are Rac and Ss, which display higher stability and even lack a distinguishable pattern. Consequently, it is necessary to transform a greater proportion of Rac/Ss into Con.

**Table 2:** Identification, proportion and separation difficulty of three types of racemates^165–168^

Identification	Racemate		
	Racemic compound (Rac)	Conglomerate (Con)	Solid solution (Ss)
Hand-sorting	Non-hemihedrism	Hemihedrism	Non-hemihedrism
SCXRD	Different from E	Consistent with E	Different from E
FT-IR, Raman and PXRD	The position of the signal is different from E	The position of the signal is consistent with E	Irregular
Melting point and solubility	Rac > Rac + a little E, Rac < Rac + a little E	Con < E and Con < Con + a little E, Con > E and Con > Con + a little E	Irregular
Solid-state CD spectra	The response signal is strictly zero for single crystal	Consistent with E (singles crystal)	Irregular
Nonlinear optical technique (SHG)	The majority of Racs: no SHG effect. (98% of Racs crystallize in the centrosymmetric space groups such as *P*2_1_/*c*, *C*2/*c*, *Pbca*, and *P*1̄)	The majority of Cons: large SHG effect. (95% of Cons crystallize in non-centrosymmetric space groups such as *P*2_1_2_1_2_1_, *P*2_1_, *C*2, and *P*1)	Irregular
Occurrence/frequency	>90%	5–10%	<1%
Relative difficulty	Medium	Easy	Hard

One approach to achieve this goal is to investigate suitable crystallization conditions (temperatures, solvent, additives, external fields, *etc.*) to enhance the thermodynamic stability of Con or inhibit the nucleation of Rac. For example, Lee *et al.* monitored the effects of higher temperature and the presence of succinic acid additives on the conversion of aspartic acid from Con to Rac. The results showed that higher temperature lowered the relative stability of Con, whereas the addition of succinic acid inhibited the formation of Rac.^151^ Additionally, baclofenium hydrogenomaleate (BaHMa) presents a partial Ss at room temperature with a high eutectic composition (98.5–100% ee), which allows facile resolution *via* auto-seeded PC. However, PC failed to resolve BaHMa in the case where the temperature was higher than 145 °C because of the formation of the complete Ss phase.^152^ Marine Hoquante and coworkers identified a novel Rac for BINOL-OBn whose melting point exceeded that of Con by 20 °C.^153^ Consequently, the initial Con crystals in a −10 °C diethyl ether suspension were eventually transformed into Rac crystals despite the addition of Con seeds.^154,155^ In addition, Białońska *et al.* compared the crystal structures of seven brucinium salts and found that anionic dimers, which hinder enantio-separation by constructing brucinium diastereomeric double salts, could be eliminated by increasing the polarity of the solvent.^156^ Another study reported a solution-free method for the preparation of Con, in which mechanochemistry processes with non-polar and polar solvents led to the formation of the Con- and Rac-salt of tartaric acid·isoniazid, respectively.^157^ Additionally, chiral molecules antiparallel to the spins of electrons in the metal substrate can exhibit enantioselective adhesion, agglomeration and crystallization. Inspired by this, Tassinari and coworkers placed magnets beneath the substrate at the bottom of a crystallizer containing either a racemic or enantiopure solution of amino acids (DL-Asp, DL-Glu·HCl, or DL-Thr). Consequently, the magnets facing the N and S directions could enantiomorphously crystallize all the amino acids on the substrate, while only DL-Asp could be resolved by both magnets.^89^ Moreover, Zhou *et al.* found that the addition of ultrasound could enable dl-glutamic acid (dl-Glu) to crystallize as Con rather than the thermodynamically stable Rac. This means that the acoustic cavitation effect can kinetically induce homochiral nucleation when the thermodynamic stability of Con and Rac is similar.^158^ Hence, carefully selecting the crystallization conditions may help influence the crystallization kinetics of Con, Rac and Ss and reduce the undesirable disappearance of Cons, in particular for chiral molecules exhibiting high molecular flexibility or lacking significant strength differences in their intermolecular interactions.^155^

**Fig. 5:**
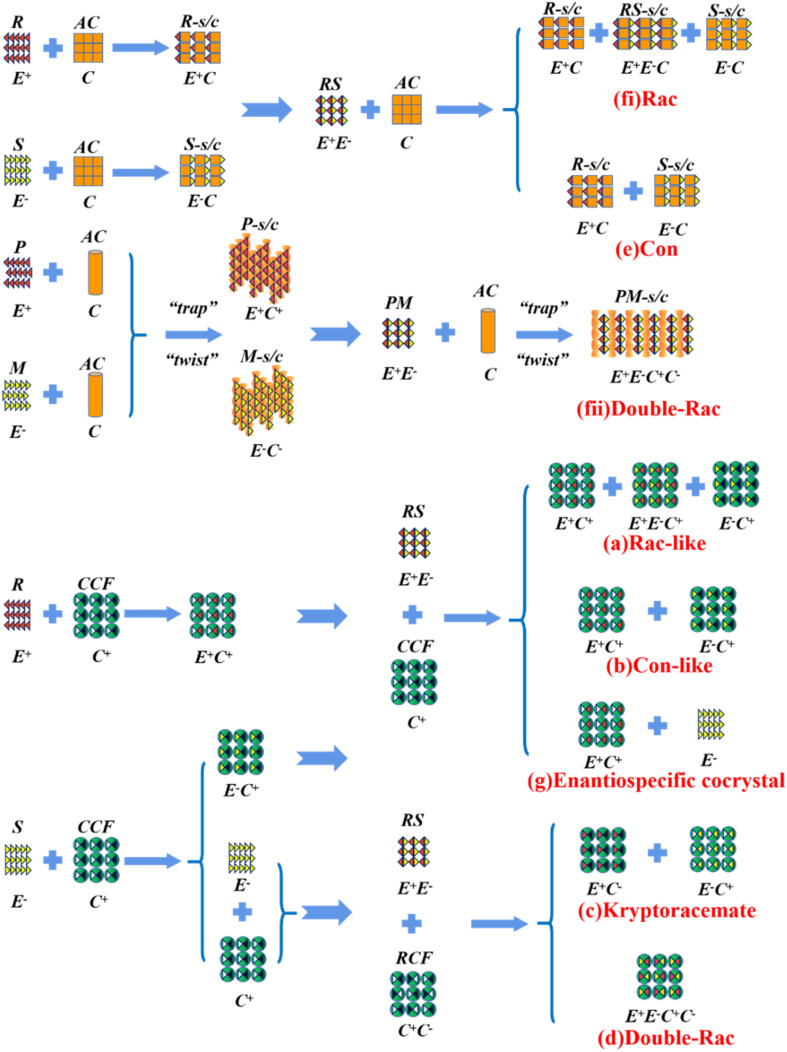
Representative outcomes of the crystallization of a racemic mixture (*RS* or E^+^E^−^) with chiral (C^+^), racemic (C^+^C^−^), and achiral (C) coformers, resulting in (a) and (b) diastereomers, (c) kryptoracemates, (d) double racemic compounds, (e) conglomerates, (fi) racemic cocrystals/salts, (fii) double racemic cocrystal/salts, and (g) enantiospecific cocrystals. E^+^: *R*-enantiomer, E^−^: *S*-enantiomer, AC: achiral coformer, CCF: chiral cocrystal coformer, RCF: racemic cocrystal coformer, *R*-s/c: *R*-salt/cocrystal, *S*-s/c: *S*-salt/cocrystal, *RS*-s/c: *RS*-salt/cocrystal, *P*-s/c: *P*-salt/cocrystal, *M*-s/c: *M*-salt/cocrystal, and *PM*-s/c: *PM*-salt/cocrystal.

Alternatively, crystal engineering provides various non-covalent interactions for chiral molecules to modify their molecular flexibility and crystal packing arrangements, and thus becomes a more potent strategy.^159^ It can be seen in Fig. 5 that introducing a chiral agent C^+^ to the racemic E^+^E^−^ molecular units can lead to the formation of Rac-like (a), Con-like (b), or enantiospecific cocrystal systems (g). However, only the formation of diastereomeric pairs or enantiospecific cocrystal benefits enantioseparation due to the disappearance of the Rac phase (Fig. 4c and g). The addition of the racemic reagent C^+^C^−^ to E^+^E^−^ may generate a double-Rac cocrystal (d), which is equally challenging to resolve as Racs, or a rare but easily resolvable kryptoracemate Con (c).^160,161^ Specially, an achiral guest C with chiral induction potential may result in novel Cons (e) and single- or double-Rac cocrystals (fi and fii). Consequently, it has become a hot topic to convert Rac/Ss into (e), (b), (g) and (c), which can be easily resolved by direct crystallization. For instance, *RS*-carvedilol remains Ss after forming salts with strong acids (hydrochloride or hydrobromide), while forming a Rac salt with a weaker acid (oxalic acid),^162^ where one oxalate anion can stably bond with one *R*- and one *S*-molecule in the crystal lattice. Similarly, oxalic acid can transform fluoxetine into Con-salt crystals, where the oxalate molecules exclusively serve as linkers between the homochiral fluoxetine cations, thereby revealing miniature fluoxetine isomers by forming homochiral 1D chains through N^+^–H⋯O^−^ interactions.^163^ However, this method does not always apply, as described in Fig. 4g. In 2023, two diastereomeric salts formed between l-DMTA and *RS*-4-cyano-1-aminoindane (4C1A) constituted a new Ss system, where the disordered *R*-isomer was found in the asymmetric unit of the *S*-4C1A:l-DMTA crystals at an occupancy of 13%. This means that *R*-4C1A:l-DMTA is less stable and is easy to dissolve. To avoid a strenuous cascade of the crystallization process, the Ss obtained from single-stage crystallization (28% ee_*S*_) underwent an enantioselective dissolution process to remove the soluble *R*-4C1A:l-DMTA salt, and crystals with final ee_*S*_ of 96% could be obtained.^164^ Compared to selective crystallization, this enrichment pathway, according to the lever rule, seems particularly efficient in Ss systems, where the tie line traversing the racemic composition has a steep slope.

**Fig. 6:**
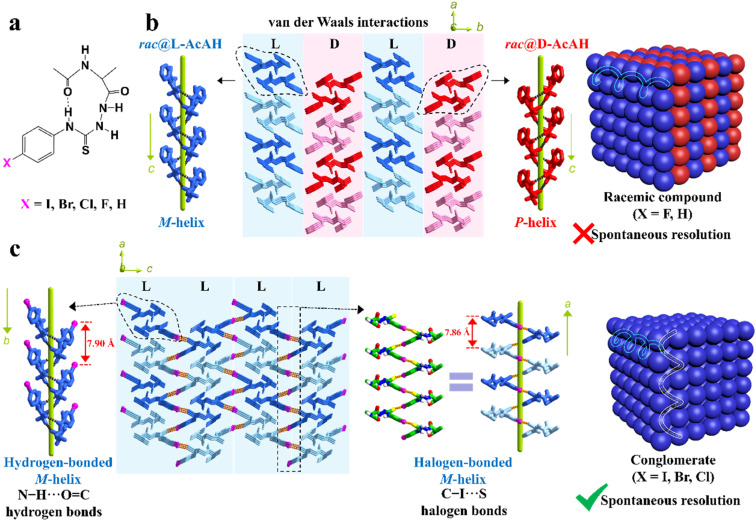
(a) Chemical structures of AcAX. (b) Heterochiral 2D layers constructed by only N–H⋯O=C hydrogen bonds and weak van der Waals interactions, which further forms 3D racemic compounds (X = F and H). (c) Homochiral 2D layers constructed by both N–H⋯O=C hydrogen bonds and C–X⋯S halogen bonds, which further form 3D conglomerates (X = I, Br, and Cl).^169^ Reproduced with permission from ref. 169. Copyright 2022, John Wiley and Sons.

Besides, the chemical derivatization strategy, which introduces new substituents or functional groups into pristine Rac/Ss, can also lead to novel Cons. In 2022, Lin *et al.* described the necessity of strong heterogeneous intermolecular interactions in a subtle but efficacious way.^169^ They elaborated on the topological structures of acetylalanine-based *N*-amidothiourea derivatives (AcAX) possessing unique substituents (X = I, Br, Cl, F, and H) (Fig. 6a). Consequently, AcAI, AcABr and AcACl underwent spontaneous resolution as Cons, while AcAF and AcAH existed as Racs. It can be seen from the crystal structures (Fig. 6c) that the 1D homochiral helices of the AcAX (X = I, Br, and Cl) molecules along the *a* axis are extended by N–H⋯O=C to form 2D layers along and *aoc* plane, followed by further stacking along and *b* axis *via* C–X⋯S halogen bonds. The dominant orientations of these two interactions remained perpendicular to each other and improved the stability of Con crystals. By comparison, the lack of halogen bonds in the crystal structures of AcAF and AcAH undermines the self-assembly preference of homochiral 2D layers, which gave way to the packing between counter chirality layers by mere van der Waals interactions (Fig. 6b). Consequently, strong heterogeneous interactions that tend to form connections between homochiral molecules (such as halogen bonds) should be specifically created for target enantiomers to “disseminate” their respective chiral signals.

To date, although there are multiple cases related to Cons, the efficient screening and prediction of these systems require significant time and have a lack of guiding principles, and scarce research has established connections between the CSD and the design of Cons.^170^ Consequently, it is important to develop fitting and predictive models considering various aspects such as functional groups, molecular flexibility, melting point, solubility, and intermolecular interactions using MD and DFT calculations. Moreover, crystal structure prediction (CSP),^171^ which considers symmetry, intermolecular interactions and energy minimization, may explore potential “polymorphs” of a racemate, *i.e.*, Con, Rac and Ss, due to the identical functional groups and connectivity of two enantiomers.

### 4.2 Preferential crystallization (PC)

Spontaneous resolution is a term describing the resolution of a racemate that crystallizes as a conglomerate.^172^ However, this theory can only generate a physical mixture of enantiomers unless their crystals exhibit significant differences in size, morphology, color, *etc.* The essential tool for the industrialization of spontaneous resolution is preferential crystallization (PC), where the target products can be mass-produced by regulating the crystallization kinetics by seeding the desired enantiomer in both batch and continuous operating mode.^173,174^ Recently, many innovative coupled setups and chiral additives with molecular similarity to enantiomers have been utilized to inhibit the nucleation of the unwanted enantiomer, enhance the productivity or satisfy special requirements, which pertains to the latest developments in PC.^61,62,175^

#### 4.2.1 Phase diagrams of preferential crystallization

**Fig. 7:**
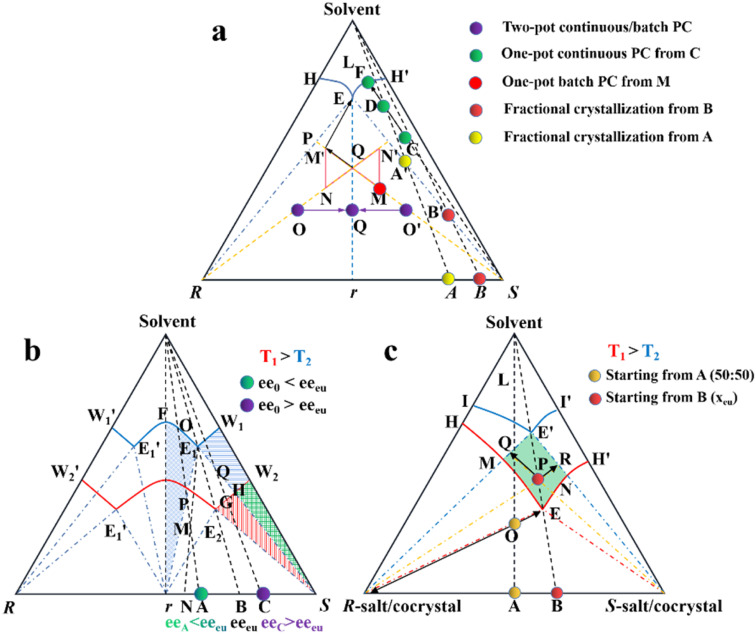
Ternary phase diagrams illustrating the equilibration and preferential crystallization of (a) conglomerate, (b) racemic compound, and (c) pair of diastereomers.

Conglomerate-forming systems can be effectively resolved through PC regardless of the initial enantiomeric excess (ee_0_). In the case where the ee_0_ of the feed lies in a two-phase region (Fig. 7a, H′ES), the addition of *S*-seeds enables the direct crystallization of optically pure *S*-crystals. Consequently, the thermodynamically equilibrated solid and liquid compositions are located at point S and the EH′ curve (point F), respectively, in a batch operation. By comparison, the mother liquor composition remains in the two-phase region in a continuous process (point D) due to the non-equilibrium nature of the operation. Alternatively, when ee_0_ falls within a three-phase region at point M (or Q), the addition of *S*-seeds in the metastable zone can promote the secondary nucleation and provide a larger crystal surface for *S*-enantiomer; thus, *S*-solute will preferably precipitate for the growth of homochiral crystals with the mother liquid composition moving from point M (or Q) to M′. Meanwhile, to avoid the spontaneous nucleation of *R*-crystals, during which the composition of the mother liquor migrates to eutectic point E, the slurry needs to be filtered before reaching point M′. After replenishing the racemate, ee_0_ can be reset at the *R*-enriched N point (or zero ee_0_ Q), allowing the alternating harvest of the two enantiomers in a single vessel batch PC. Notably, two-pot PC setups with liquid coupling can achieve higher productivity and scale-up ability under a specific circulation rate. After seeding, the liquid composition within two connected containers will gradually approach the steady state from point O and O′ to point Q for a continuous process, while that for a batch process will constantly remain at point Q.

Considering that Con accounts for a maximum of 19% of the racemates, expanding the application of PC to Rac is of great significance (Fig. 7b).^176^ In general, PC is feasible for Racs with higher solubility than their pure enantiomer and an ee_0_ higher than the ee value at the eutectic point (ee_eu_).^177,178^ In the case where ee_0_ < ee_eu_, the maximum ee of the product is simply ee_eu_. For the slurry composition initially located in the ΔE_1_Br region (point M starting from point A), ee_filtrate_ = ee_eu_ > ee_0_, ee_re*s*idue_ < ee_0_ < ee_eu_, while that in the fan-shaped FE_1_r area (point P starting from point A) is 0 ≤ ee_filtrate_ ≤ ee_eu_, ee_re*s*idue_ = 0. Alternatively, when ee_0_ > ee_eu_ (starting from point C), the smallest amount of solvent and the highest yield of *S*-crystals can be achieved at point Q. In addition, point H located at the boundary of the two-phase zone E_2_W_2_S (*T*_1_) will be affiliated with the three-phase zone E_1_BS as the temperature decreases (*T*_2_). This accounts for the precipitation of Rac when the temperature drops below the threshold, and thus both the initial composition and temperature range should be carefully selected. Fortunately, the addition of chiral additives (Section 4.2.3) can provide a window to delay the appearance of Rac although the operation needs to start at an ee_0_ somewhat higher than ee_eu_.

Additionally, the crystallization process of diastereomeric cocrystals/salts closely resembles that of enantiomers (Fig. 7c). When the initial composition lies at point A (*R*-diastereomer : *S*-diastereomer = 50 : 50), the maximum yield of pure *R*-diastereomer can be obtained through the direct crystallization in the two-phase region (point O, *T*_1_), with the *S*-diastereomer-enriched mother liquor located at point E. Ingeniously, Simon and coworkers transferred half of the mother liquor into another crystallizer and coupled it with the original crystallizer *via* crystal-free liquid-phase exchange. The simultaneous preparation of two diastereomers in the metastable zone ME′NE (*T*_2_) could be achieved in this coupled batch setup, which is attributed to the addition of *S*-seeds to the new crystallizer and continued growth of *R*-crystals in the original vessel. Similarly, compositions of the liquid phase in two tanks would approach points R and Q, respectively, and finally end up located at point E′. This coupled setup not only maintained the purity of the *R*- and *S*-diastereomer (*ca.* 99%), the productivity of the *R*-diastereomer, and the yield of the *S*-diastereomer (*ca.* 20.5%), but also doubled the productivity of *S*-crystals and achieved 49% yield for *R*-crystals, all with just an additional one-third of the operating time.^179^ Hence, this strategy is particularly promising for cases where the desired enantiomer forms a more soluble salt or a large crystal size is desired.

It can be observed that regardless of the symmetry of the phase diagram, a crystal-free liquid coupling strategy for “PC” within the three-phase region may significantly lower the overall supersaturation and promote the growth of homochiral crystals, thereby reducing the contamination by impurities, while enhancing the productivity and yield. Other variants with liquid-phase exchange are introduced in Section 4.2.2.

#### 4.2.2 Variants of preferential crystallization

**Fig. 8:**
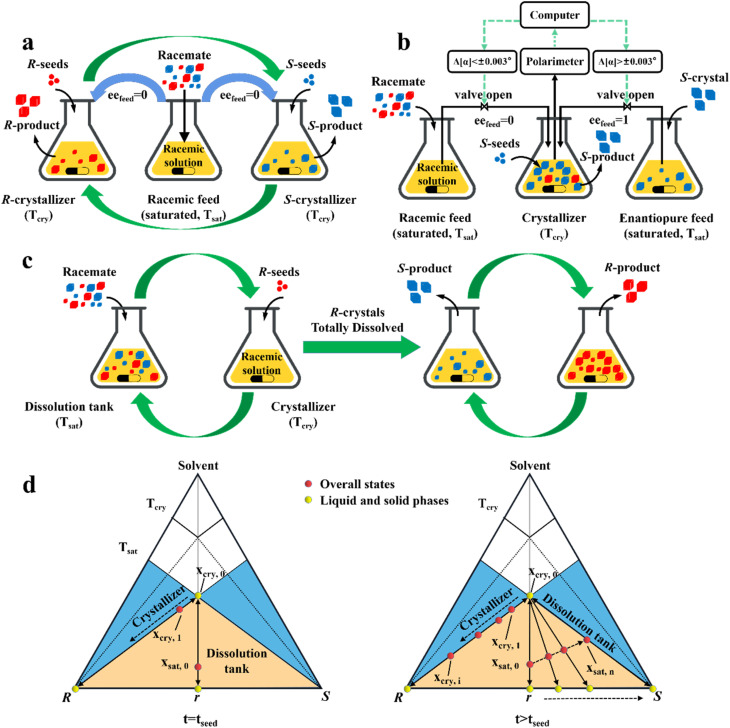
Two-pot variants of preferential crystallization. (a) Coupled continuous/batch preferential crystallization (CPC) process. (b) CPC process with temporarily controlled feed composition. Δ[*α*]: change in optical rotation value of the liquid phase. (c) Coupled preferential crystallization-dissolution (CPC-D) process. (d) Representation of the composition evolution of the saturation tank and crystallizer in the ideal CPC-D process at the time of seeding (left) and after seeding (right). Both blue and green bent arrows indicate crystal-free liquid exchange. The supersaturation (Δ*T* = *T*_*s*at_ − *T*_cry_) is incapable of triggering primary nucleation.

In traditional PC, a too-short induction period and a slightly delayed experimental endpoint may trigger the nucleation of the counter-enantiomer, resulting in reduced productivity. Hence, there is a growing demand for variants to address this issue. For example, a first-generation device is coupled with continuous/batch preferential crystallization (CPC).^61,62^ This combination decouples the non-enantioselective crystallization of two enantiomers into two separate tanks through a crystal-free liquid circulation (Fig. 8a and 7a (O, O′ → Q)), which can reduce the instant supersaturation level and the nucleation/growth rate of the undesired enantiomer despite the uncertainty of the endpoint.^62,180–182^ Based on CPC, Thomas Vetter and coworkers installed heated mills before and after the two crystallizers to *in situ* generate seeds by dissolving fine crystals. Further scaling up the process offers higher efficiency and lower solvent usage compared to one-pot operation despite the complex and sensitive consideration of the scaling factor.^183^ Moreover, the PC setup with an additional feed tank can also conveniently control the ee value of the product by *in situ* polarimeter monitoring (Fig. 8b). Specifically, the system will alter the feeding source from the racemate to *S*-enantiomer once the undesired *R*-crystals appear, that is, when the optical rotation value ([*α*]) of the steady-state solution varies; otherwise, the inlet tube will be switched back to zero ee_feed_.^63^ This controlled system enables high reproducibility and operation at high supersaturation levels without the need to consider the nucleation and growth kinetics because the *R*-enriched mother liquor can continuously leave the crystallizer. Interestingly, although the stability of this continuous operation is at the expense of consuming a portion of the existing *S*-enantiomer, or in other words, the precipitation of *R*-chiral impurity crystals is “hidden” due to the addition of an equal amount of *S*-seeds, replacing control of the crystallization kinetics with intuitive operational conditions is an important consideration.

In addition, a more cost-effective process, coupled preferential crystallization-dissolution (CPC-D), was developed for substrates without accessible enantiopure seeds and sufficient solubility data (Fig. 8c). Its most significant feature is that the dissolution tank contains suspended solid crystals throughout the whole process. Specifically, upon the addition of *R*-seeds, the *R*-enantiomer in the liquid phase will be unsaturated in the dissolution tank because of the growth of *R*-crystals in the crystallizer. Therefore, the *R*-enantiomer in the suspension will be preferentially dissolved to maintain its saturation state in the liquid phase at *T*_sat_, retaining *S*-crystals in the dissolution tank. When all the *R*-crystals in Con are completely dissolved and unable to replenish the required supersaturation in the crystallizer, the operation reaches the endpoint. Notably, the selective dissolution rate of *R*-molecules should be higher than that of their crystallization and the liquid circulation rate should be sufficient. This is further illustrated in Fig. 8d, where the overall state in the crystallizer changes from the equilibrated liquid-phase composition, *x*_cry,0_, to *x*_cry,1_, while the total composition in the dissolution tank remains in excess at *x*_sat,0_ (Fig. 8d, left). Subsequently, the continuously replenished racemic solution in the crystallizer causes the overall operating point to gradually shift to the *R*-vertex along the material balance line. Correspondingly, the *R*-enantiomer that is continuously dissolved from the Con crystals enables the liquid operating point in the dissolver to gradually move from *x*_sat,0_ to *x*_sat,*n*_, accompanied by the composition of the solid phase from 0% (point *r*) to 100% ee_*S*_ (*S*-vertex) (Fig. 8d, right). Notably, the quality of the obtained *R*-crystals will be higher than that of their antipode due to the condition of *T*_sat_ > *T*_cry_ (Fig. 8c). Inspired by this method, Hein *et al.* utilized slightly enantiomeric excess to selectively control the competitive crystal growth of omeprazole enantiomers. By operating two vessels in series and using a temperature gradient as the driving force, *S*-omeprazole with a chiral purity of higher than 98% was harvested.^64^ Also, Eicke *et al.* compared the productivity and purity between CPC-D and CPC processes.^181^ Under the same initial conditions, they both exhibited close values for productivity and 100% ee in the first 15 min. However, CPC exhibited a sharp rise in supersaturation consumption rate afterward, affording the two enantiomers of threonine in their respective tanks with a productivity of 13 g L ^−1^ h^−1^. By comparison, CPC-D displayed better productivity of 22 g L ^−1^ h^−1^ in the crystallizer although the enantiopurity in the dissolution tank was slightly lower than 99% ee.^184^ Additionally, a scaled-up CPC-D setup, which consisted of two double-walled 450 mL tanks, achieved the resolution of (*RS*)-guaifenesin with good yield and ee values of *y*_*R*_ = 22.5%, ee_*R*_ = 95% and *y*_*S*_ = 47.5%, ee_*S*_ = 80% in the crystallization tank and dissolution tank, respectively.^185,186^ Hence, CPC-D can serve as a countermeasure when only one seed is accessible, although it may lead to somewhat lower productivity in the crystallization tank than that in single-vessel batch PC.

#### 4.2.3 Reverse preferential crystallization

**Fig. 9:**
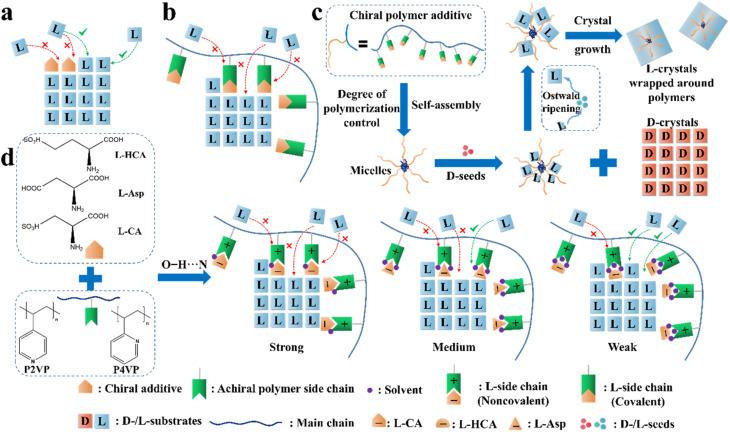
Illustration of the application of the “rule of reversal” in PC. Mechanism for the adsorption of (a) “tailor-made” additives and (b) “tailor-made” covalent polymeric additives on the growing face of l-crystals. (c) Graphical illustration of the resolution process in the presence of both d-seeds and chiral polymer inhibitors.^65^ (d) Mechanism for the adsorption of noncovalent additives on the growing face of l-Thr.^66^

The “reverse PC strategy” is a new method that has emerged recently based on the “rule of reversal” (“rule”).^187^ It refers to the phenomenon where the addition of “tailor-made” chiral additives leads to the preferential crystallization of the enantiomer of opposite chirality to the seeds. This results from the molecular similarity, such as comparable functional groups, interactions and packing modes, between the chiral additive molecules and a specific single enantiomer. The additive molecules will be selectively adsorbed on the crystal surfaces of this enantiomer, and in most cases, hinder its further crystal growth due to the occupation of the growth sites and the incongruent self-assembly of the side chains of the additive molecules (Fig. 9a). Therefore, this process is a combined result of seed-promoted PC of the target enantiomer and additive-inhibited crystallization of the counter-enantiomer. For example, in 2022, Lu *et al.* utilized (*R*)- or (*S*)-1,3-butanediol as an additive to realize PC of an Rac, dl-citrulline (ee_eu_ = 87%). The MD and molecular electrostatic potential simulations results indicated that the *R*-additive molecules established more prominent interactions with d-citrulline than the l-configuration, whose nucleation kinetics was more significantly enhanced. Hence, the addition of 2% l-citrulline seeds and 0.5% (*R*)-1,3-butanediol achieved 25.4% yield and 99% ee_L_ from a citrulline solution (ee_0_ = 88%).^188^

Another method is loading chiral additives on a polymer. Similarly, the enantioselective incorporation of the l-molecules in the polymer arms can enhance the possibility of enantioselective absorption due to the evenly distributed chiral recognition sites in a folding structure (Fig. 9b), thereby enlarging the metastable zone and disrupting the nucleation and crystal growth of l-crystals. For example, Ye *et al.* copolymerized a T-shape l-lysine-based polymer (PMAL) with the main chain of tri(ethylene glycol)-grafted polymethylsiloxane (PMS-*g*-TEG), followed by further anchoring a fluorescent agent on the end of the T-shape part to fabricate a micelle structure with PMAL as the arms and PMS-*g*-TEG as the core (Fig. 9c).^189^ Expectedly, small colorless d-Asn·H_2_O crystals fully crystallized within the first 14 h (99.9% ee and 16.9% yield) after adding the polymer to a racemic asparagine monohydrate (Asn·H_2_O) solution, followed by the precipitation of large red polymer-wrapped l-Asn·H_2_O crystals (99.5% ee and 13.8% yield). Another Con, threonine enantiomers, underwent a similar enantioseparation process with >98.4% ee and >15.5% yield. The size differences in both cases indicate that the trapped l-enantiomers experienced an Ostwald process, where the inhibited l-crystals on the branches grew slowly and enclosed the micelle. In 2021, this group anchored chiral molecules on the backbone of achiral polymers through non-covalent interactions and prepared a series of supramolecular polymer inhibitors (Fig. 9d).^66^ Three l-chiral acids were employed individually to form hydrogen bonds with two achiral polymers, P2VP and P4VP, through O–H⋯N interactions. Between them, the polymer composed of l-CA and P4VP exhibited the highest enantioselectivity toward l-Thr due to the highest binding energy between the l-CA molecule and the main growing face of the l-Thr crystal, and the intense anion–cation interaction between l-CA and P4VP. Hence, the l-CA pendants could be strongly curved around the main chain and displayed the highest inhibition effect. It is foreseeable that non-covalent additives can also be tailored for the preparation of metastable Racs such as glutamic acid and nimodipine, which will broaden the application prospects of the reverse PC strategy. Interestingly, the same group enclosed Fe_3_O_4_-based magnetic nano-splitters in amphiphilic polymeric additives, which resulted in the formation of l-crystals with magnetism.^190^ Subsequently, the l-crystals could be easily separated from the solution and the nano-splitters could be recovered by simply dissolving the products in water and using a magnet. Consequently, a yield of 95.1% was achieved with enantiopurities of over 95.0% for both enantiomers.

Although PC is highly economical and can provide a decent yield and ee, the capricious entrainment of the unwanted antipode and the limited amount of Con are two significant challenges. Fortunately, the growing field of Con systems has strengthened the substrate scope of PC,^65,66^ and the chiral contamination can be mitigated *via* the development of generic PBMs including both simple PBMs for quick prediction and precise PBMs for process design. More importantly, devising variants such as multi-vessel liquid-phase exchange setups (CPC, CPC-D),^61–64^ coupling processes (deracemization^60^ and membrane^191^) and reverse PC (chiral additives) can inhibit the crystallization kinetics of the undesired enantiomer, thereby improving the productivity, enantioselectivity and process stability.

### 4.3 Preferential enrichment

**Fig. 10:**
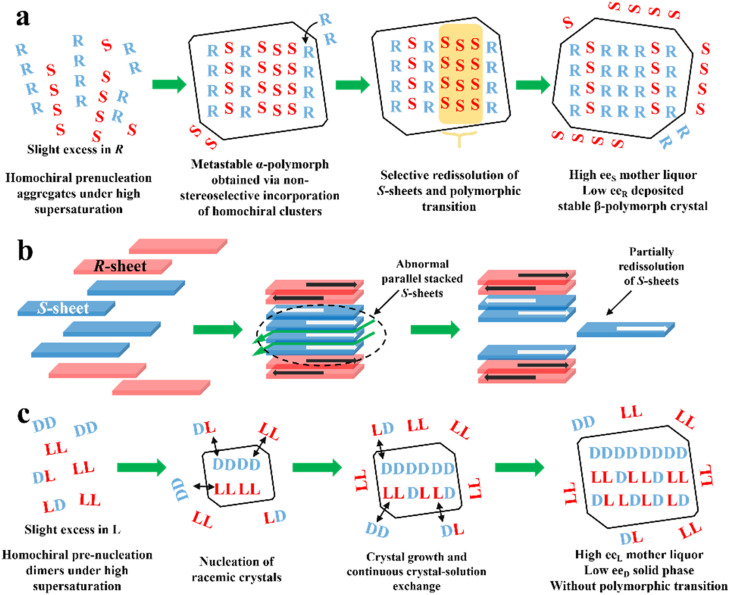
(a) General mechanism of PE for a slight *R* excess first-generation organic compound.^197^ (b) PE mechanism of CPPPA·INA. The green arrows between layers indicate the brittle crystal sites.^194^ (c) PE mechanism of the dl-Arg·fumaric acid cocrystal.^198^

PE is technology utilizing static, far-from-equilibrium crystallization to resolve racemic mixtures, especially Rac and Ss. The slightly excess enantiomer in Rac/Ss with a certain ee_0_ (≈5%) can be preferentially enriched in the mother liquor with a high ee value (≈ee_eu_), while the non-excess enantiomer becomes somewhat dominant in crystals (−2 to −5% ee). The classical PE process suitable for the preparation of first-generation racemates^192,193^ involves the following steps (Fig. 10a): (I) high supersaturation of the racemate (4–25 fold) and considerable solubility difference between the enantiomers and racemate (at least 2-fold) initiate the formation of homochiral associations/assemblies such as 1D chains or 2D sheets; (II) the nucleation aggregates or clusters undergo non-enantiospecific incorporations to form metastable Ss crystals; (III) homochiral chains/sheets are selectively redissolved from metastable crystals, which transforms the crystals into a more stable polymorph *via* solvent-assisted polymorphic transition; (IV) the slightly excess enantiomer in the original Rac/Ss can be preferentially enriched in solution with high ee, with its antipode deposited in low ee crystals (Fig. 10a). Therefore, this symmetry-breaking process can be seen as a violation of stable equilibria because extremely high supersaturations allow the composition of the crystals and mother liquor to deviate from the tie-line trajectory and equilibrium.^194^ Considering kinetics and thermodynamics, Uchida *et al.* reported that simply repeating crystallization-redissolution at high supersaturation could facilitate the phase transition and nonlinear solubility of two enantiomers.^195^ In addition, Takahashi *et al.* described the above-mentioned procedure by dynamically monitoring the structural changes in stable and metastable crystals during PE.^196^ However, the guiding principles for producing a mother liquor with a composition of ee_eu_ through this equilibrium-deviated process still remain unclear.

To expand the candidate scope from first-generation racemates, particularly organic sulfates, to other species, amino acids and ketoprofen were selected as second-generation compounds to undergo the PE process. Interestingly, these substrates exhibit the PE phenomenon but fail to undergo solvent-mediated polymorphic transformation, and thus questions arise regarding the necessity of condition (III).^199^ For the same purpose, the crystal engineering strategy (Fig. 5a and fi) are employed to transform low ee_eu_ Racs into higher ones or promote the probability of polymorphic transformation by introducing coformers. For example, dl-Phe·fumaric acid, dl-Arg·fumaric acid and dl-leucine·oxalic acid exhibited the PE phenomenon after cocrystallizing with achiral coformers.^197,200^ Among them, the ee_eu_ of dl-leucine (Leu) experienced a significant increase from 19% to more than 98% after forming the dl-leucine·oxalic acid cocrystal, which facilitated an enantio-enrichment of 45% ee_L_. This is because the formation of the cocrystal enhanced the binding affinity of the homochiral Leu molecules and the l-dominant pre-nucleation aggregates were transformed into crystals, where the 2D l-sheets outnumbered the d-sheets, enabling the redissolution of the redundant l-sheets. In 2019, Takahashi *et al.* endowed 2-(4-((4-chlorophenoxy)methyl)phenoxy) propionic acid (CPPPA) with the potential for PE by cocrystallizing it with isonicotinamide (INA). The new Rac cocrystal CPPPA·INA resulted in an optical purity of up to 93% ee_*S*_ in the mother liquor.^194^ Notably, the two adjoining homochiral layers interacted with each other by C–H⋯O, while only weak C–H⋯π interaction remained between the heterochiral layers. Accordingly, the cocrystal-induced differential interactions between the chiral layers led to the preferential dissolution of homochiral layers. In general, a large dipole moment arrays the homochiral molecular layers in an anti-parallel mode. However, under high supersaturation conditions, part of the homochiral layers exhibit less compact stacking at the interface, which triggers partially, rather than completely, the redissolution of the irregularly parallel stacked (*S*)-sheets (Fig. 10b). In this novel mechanism, the considerably enhanced dipole moment of CPPPA by the cocrystal and weak interactions between the homochiral layers (C–H⋯π and C–H⋯O) proved to be responsible. Thus, the crystal engineering strategy can impart Racs with unique intermolecular interactions, packing modes and polymorphism, leading to a different PE mechanism. In 2023, the recapture of a second-generation cocrystal, dl-Arg·fumaric acid, further raised doubts about the necessity of the polymorphic transformation. Interestingly, in the sequential washing liquid of the collected crystals from the l-Arg excess starting material, the outer crystal layer (≈15 wt%) possessed about 30% ee_L-Arg_, while the interior parts (≈80 wt%) consisted of 6% ee_D_ molecules. Furthermore, with the addition of 10 μL ^13^C_6_-labeled L-Arg enantiomer solution (0.1 wt% of the total dl-Arg), despite the completion of PE, ^13^C_6_ labeled molecules could still be found in the crystal phase.^198^ These results showed that PE of dl-Arg·fumaric acid is only driven by the material exchange between the solution and crystals under high supersaturation, rather than polymorphic transition (Fig. 10c).

These cases show that PE allows for small-scale resolution for Racs and serves as a potent tool to understand the origin of chirality. However, it still faces challenges such as limited applicability and stringent prerequisites, lack of guiding principles for process design, and inconsistent mechanisms. Also, the Ss phase is an important intermediate state for both the polymorphic transition and selective dissolution. Unfortunately, little research has been done regarding the resolution of racemic Ss using PE. Besides, it seems that solvent-mediated polymorphic transformation is not necessary in PE and it remains ambiguous whether previously reported polymorphic transition cases are just special consequences of Ostwald ripening. Hence, these issues can hinder the application of this rudimentary theory to other Rac/Ss. Interestingly, more cocrystals of Racs such as dl-Phe·fumaric acid, dl-Arg·fumaric acid and dl-leucine·oxalic acid exhibited PE phenomena.^194,198^ Hence, the crystal engineering strategy may enlarge the substrate scope of PE and contribute to its the exploration of its mechanism.

### 4.4 Chemical resolution

According to the type of product obtained by the “reaction” between the chiral reagent and the racemate, the chemical resolution method can be divided into kinetic resolution, classical chemical resolution, cocrystal-based resolution, and Dutch resolution.

#### 4.4.1 Kinetic resolution

**Fig. 11:**
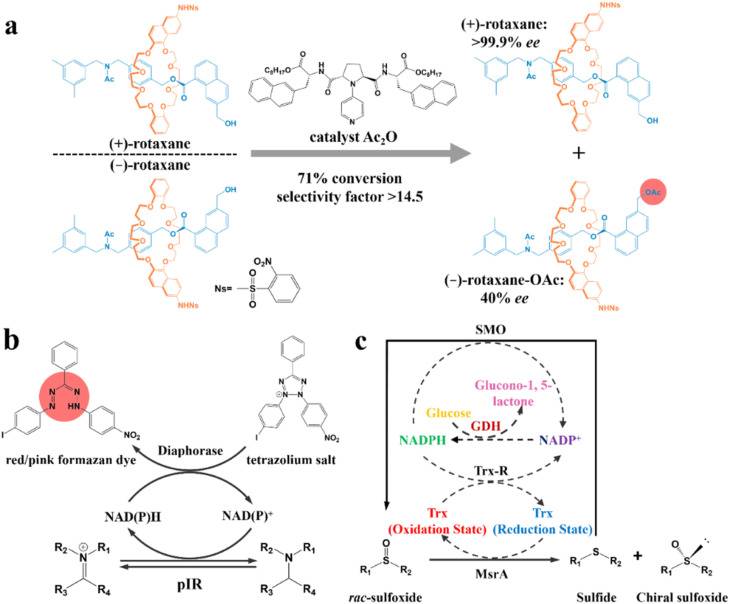
(a) Kinetic resolution of racemic mechanically planar chiral rotaxane.^201^ (b) Mechanism of high-throughput enzyme screening based on a dye.^202^ (c) Schematic representation of the multienzyme cascade cyclic deracemization system with cofactor regeneration for the preparation of chiral sulfoxides. Dashed arrows represent the cofactor regeneration cycles.^204^

KR relies on the disparate catalytic kinetics of a chemical regent or enzyme toward enantiomers. For example, Kawabata and coworkers successfully disengaged racemic mechanically planar chiral rotaxane molecules through enantioselective acylation of the remote –OH group of (−)-molecules (Fig. 11a). The selectivity was attributed to the property of the Ac_2_O-based catalyst, where a certain distance was maintained between its active site and chiral element. Furthermore, the enantioselective recognition and resolution performance could be enhanced as the acidity of the terminal substituents grafted on the naphthyl increased. Consequently, the use of an acidic macrocyclic terminal (–NHNs) achieved a separation factor of >14.5 and ee value of >99.9%.^201^

In recent years, enzymatic transformation has shown promising potential for KR with better biocompatibility, higher selectivity and milder reaction conditions compared to the use of expensive and toxic chemical reagents. Accordingly, many studies have focused on enzyme screening and structural modification. Turner and coworkers pioneered a metagenomic biocatalytic toolbox for high-throughput colorimetric screening toward imine reductases. A 384-well plate was used for characterizing the conversion rate (color intensity) and separation performance (the number of enzyme hits). Specifically, formation of the colorful formazan dye could be generated through the oxidation of the target amine, during which cofactors NAD(P)H and NAD(P)^+^ in the diaphorase-related reduction reaction will undergo an interconvertible regeneration (Fig. 11b). Consequently, the as-screened pIR-241 or pIR-358 enzyme witnessed a preparative-scale resolution with 28.5–46% yield and considerable de value of 98%.^202^ The screening method can further ameliorate the ineffectiveness of the sequence identity design, despite the requisite that enzymes should possess thermostability and the ability to drive the pigment preparation reaction. Additionally, the structural modification of enzymes can also contribute to a higher conversion rate for KR. For instance, the catalytic triad S132–Y145–R149 in the pristine halohydrin dehalogenase (HheC) crystal structure can hardly hydrogen bond the O atom of the target (*R*)-4-chloro spiro-epoxyoxindole because of the Cl atom-induced steric hindrance. However, two potential mutable parts, N176 and T134, which were recognized *via* molecular docking simulation, can replace the ineffective part in the catalytic triad. Consequently, the 2-H6 (N176A/T134I)-modified enzyme could harvest unreacted (*S*)-spiro-epoxyoxindole with 93% ee and 35% yield, while generating a new (*R*)-azidolytic product with 90% ee and 45% yield.^203^

Another novel spotlight concerns the cascade designs involving cofactor regeneration to achieve chiral amplification, which couples enzymatic KR with racemization. Peng and coworkers pioneered a multienzyme cyclic deracemization system for various sulfoxide enantiomers (Fig. 11c), where the highly enantioselective reduction of (*S*)-sulfoxides and non-selective oxidation of as-reduced sulfides can be achieved by methionine sulfoxide reductase A (MsrA) and styrene monooxygenase (SMO), respectively. Notably, the introduction of two additional enzymes, Trx reductase (Trx-R) and glucose dehydrogenase (GDH), ingeniously facilitated the regeneration of cofactors through four cycles, and thus rejuvenated the catalytic performance of MsrA and SMO. Hence, the individual KR can be integrated into a multienzyme cascade process and upgraded to a deracemization process. Surprisingly, this system accommodated one thio-alkyl, two heteroaromatic, two alkyl, and nine aryl sulfoxides, boasting the ee values and yields to higher than 90% for all the substrates. Particularly, a eutomer with 84% isolated yield and 99% ee was afforded on a large-scale (0.75 g, 1 L).^204^

Considering KR processes with milder reaction conditions, recyclable resolving agents and comparable enantioselectivities, exploratory research involving chemoenzymatic or multienzyme cascade processes has become the mainstream. However, the availability of suitable enzyme candidates that can support the cyclic utilization of cofactors still remains scarce. Given that the mechanism of KR based on novel asymmetric catalysts has been fully revealed in numerous excellent reviews,^205–207^ it will not be elucidated here.

#### 4.4.2 Classical chemical resolution

CCR is a widely used method due to its simple equipment, broad applicability, high productivity and enantioselectivity. It usually includes selecting an appropriate chiral resolving agent to form diastereomeric salts with the racemate, separating diastereomers using differences in physical properties (melting point, solubility, vapor pressure, *etc.*), and removing the resolving agent using strong acids/bases to obtain the target enantiomer. A suitable resolving agent (cheap, renewable and optically pure) usually leads to easy-to-form diastereomers, easy-to-release original enantiomers and sufficient physical property differences between diastereomers. In recent years, many excellent CCR cases have been reported based on the molecular mechanism (Fig. 4g and 5b and c) and potential operating modes (Fig. 7c and 8) described in the preceding part (Table 3).

**Table 3:** The structures of common racemates and their chiral resolving agents

Racemates	Type	Resolving agent	Optical purity	Ref.
3,5-Bis(trifluoromethyl)-α-methyl-*N*-methylbenzylamine	Rac	l-Mal	Diastereomeric excess (de) > 99%	26
			ee > 99.9%	
Sibutramine	Rac	l-DMTA and d-DBTA	ee = 98.6%	208
TpW(NO)(PMe_3_)(η2-benzene)	Con	l-DBTA	de = 96%	209
Venlafaxine	Rac	l-DTTA	ee = 99.1%	210
NLG919	Rac	d-DTTA	ee > 99%	211
3,3,3-Trifluorolactic acid	Rac	(*S*)-Phenylglycinol	ee = 99%	212
Ibuprofen	Rac	l-Lysine	ee_n-*s*alt_ = 99.2%	179
			ee_p-*s*alt_ = 98.5%	
Lamivudine	Rac	(*S*)-Mandelic acid	—	213
Phenylglycinol	Rac	[B(l-Tar(NHPh)_2_)_2_]^−^	ee = 95%	214
Phenylpropylamine	Rac		ee = 91%	
Valsartan	Rac	Dehydroabietylamine	ee = 99.1%	215

In addition, new developments have been utilized to enhance its performance.^25^ For instance, among the four diastereomeric salts formed between (*R*,*S*)-tetramisole [(*R*,*S*)-TET] and d-DBTA, the diastereomer [(*R*)-TET]_2_·[d-DBTA] exhibited the lowest solubility and is more likely to nucleate than others *via* sonocrystallization. Products with 70% ee could be harvested in DCM/H_2_O using a 4.3 W intensity within 15 min.^216^ Similarly, Ge *et al.* successfully enantio-separated (*R*,*S*)-bupivacaine *via* an ultrasonic-assisted CCR process by adopting 12,4-dinitrodehydroabietic acid (12,14-dinitroDHAA) as the resolving agent.^217^ After utilizing a Box–Behnken design with response surface methodology, the optimal conditions were obtained to prepare the (*S*)-enantiomer with 69.8% ee and 87.5% yield. In addition, PBMs encompassing simplified crystallization kinetics and objective functions can also be applied to CCR for the prediction of temperature, concentration, or particle size distribution in the system.^218^ In short, these integrating advances show a bright future to produce CCR products with higher optical purity, yields and desired size distribution.

Although CCR remains the most widely used method for enantioseparation, there is still an extensive reliance on strong organic acids/bases as chiral resolving agents. This dependence complicates the liberation of eutomers and renders the process less environmentally sound due to the utilization of stronger acids/bases. Thus, to overcome these shortcomings, CBR and DR were proposed as upgraded versions of CCR, offering improved features and capabilities.

#### 4.4.3 Cocrystal-based resolution

In CBR, traditional chiral resolving reagents such as strong acids/bases are replaced by coformers. This means that enantioseparation merely relies on non-covalent interactions between the chiral molecule and coformer, rather than salt formation reactions and ionic bonds. Moreover, CBR is more suitable for difficult-to-salinize compounds and boasts a higher probability of inducing enantiospecific recognitions (Fig. 5g). Hence, both chiral and achiral coformers play important roles in chiral resolution.

##### 4.4.3.1 Achiral coformers (AC)

The purpose of adopting achiral coformers is to convert Rac/Ss into Con for PC process (Fig. 5e) or produce a new Rac with higher eutectic composition (ee_eu_) or polymorphism suitable for PE (Fig. 5fi and fii). For example, Neurohr *et al.* used compressed CO_2_ as an anti-solvent during the cocrystallization of nicotinamide and *RS*-naproxen.^219^ The results showed that the rapid introduction of CO_2_ led to the formation of a Con, while a slower CO_2_ feeding rate promoted the formation of a Rac. This is because the instant supersaturation has an impact on the packing mode of nicotinamide in the crystal lattice. Similarly, (*RS*)-mandelic acid can be transformed to Con by forming a cocrystal with nefiracetam. Hence, nefiracetam·*R*-mandelic acid with an optical purity of higher than 98% can be obtained *via* a seeded isothermal PC process.^27^

Ionic cocrystals (ICCs) belong to a class of multicomponent crystalline solids composed of neutral organic molecules and salts in a defined stoichiometric ratio.^220–225^ In recent years, cocrystallizing Racs with metal ions has increased the scope of Cons. For example, cocrystals formed between *RS*-etiracetam (ETI) and ZnCl_2_ showed a reversible “switch” from Rac (*RS*-ETI_2_·ZnCl_2_) to Con (*S*-ETI·ZnCl_2_) when the ZnCl_2_/*RS*-ETI ratio varied from 1 : 2 to 1 : 1.^226^ This is because Rac involves stable *R*_2_^2^ (8) rings connecting heterochiral molecules, while interactions in Con can only form homochiral 2D zig-zag chains. Another example revealed the effect of the halide counterion on Con formation in which dl-histidine·LiI and dl-proline·LiBr serve as Cons, while dl-histidine·LiCl, dl-histidine·LiBr, and dl-proline·LiI serve as Racs.^227^ Similarly, Shemchuk *et al.* found that *RS*-oxiracetam (*RS*-OXI) formed a Con (*R*/*S*-OXI·MgCl_2_·5H_2_O) with MgCl_2_, while an Rac (*RS*-OXI·CaCl_2_·5H_2_O) with CaCl_2_.^228^ This can be attributed to the fact that the coordination of heterochiral OXI molecules with Mg^2+^ is not sufficient compared to that with Ca^2+^.

##### 4.4.3.2 Chiral coformers (CCF)

According to the binding affinity between CCF and enantiomers, all the possibilities involved can be summarized as follows (Fig. 5): (i) If CCF-E^+^(or E^−^) ≈ CCF-Rac ≈ E^+^–E^−^, the chance of forming an undesirable racemic Ss will increase. (ii) If CCF-E^+^(or E^−^) ≤ CCF-Rac and CCF-E^+^(or E^−^) ≤ E^+^–E^−^, the optimal result is forming a new Rac with modified ee_eu_ suitable for PE or PC (Fig. 5a). However, the enhanced resolution potential is attributed to “cocrystal formation” rather than the “chirality” of the conformer. (iii) If CCF-E^+^(or E^−^) > CCF-Rac and CCF-E^+^(or E^−^) > E^+^–E^−^, Con-like diastereomeric cocrystal pairs can be obtained (Fig. 5b and c). (iv) If both CCF-E^+^ ≫ CCF-E^−^ and the conditions in (iii) are satisfied, the ideal enantiospecific systems can be obtained (Fig. 5g). Herein, given that the majority of reported cases are based on (iii) and (iv), novel and intriguing phenomena related to these two conditions are elaborated in this section.

**Fig. 12:**
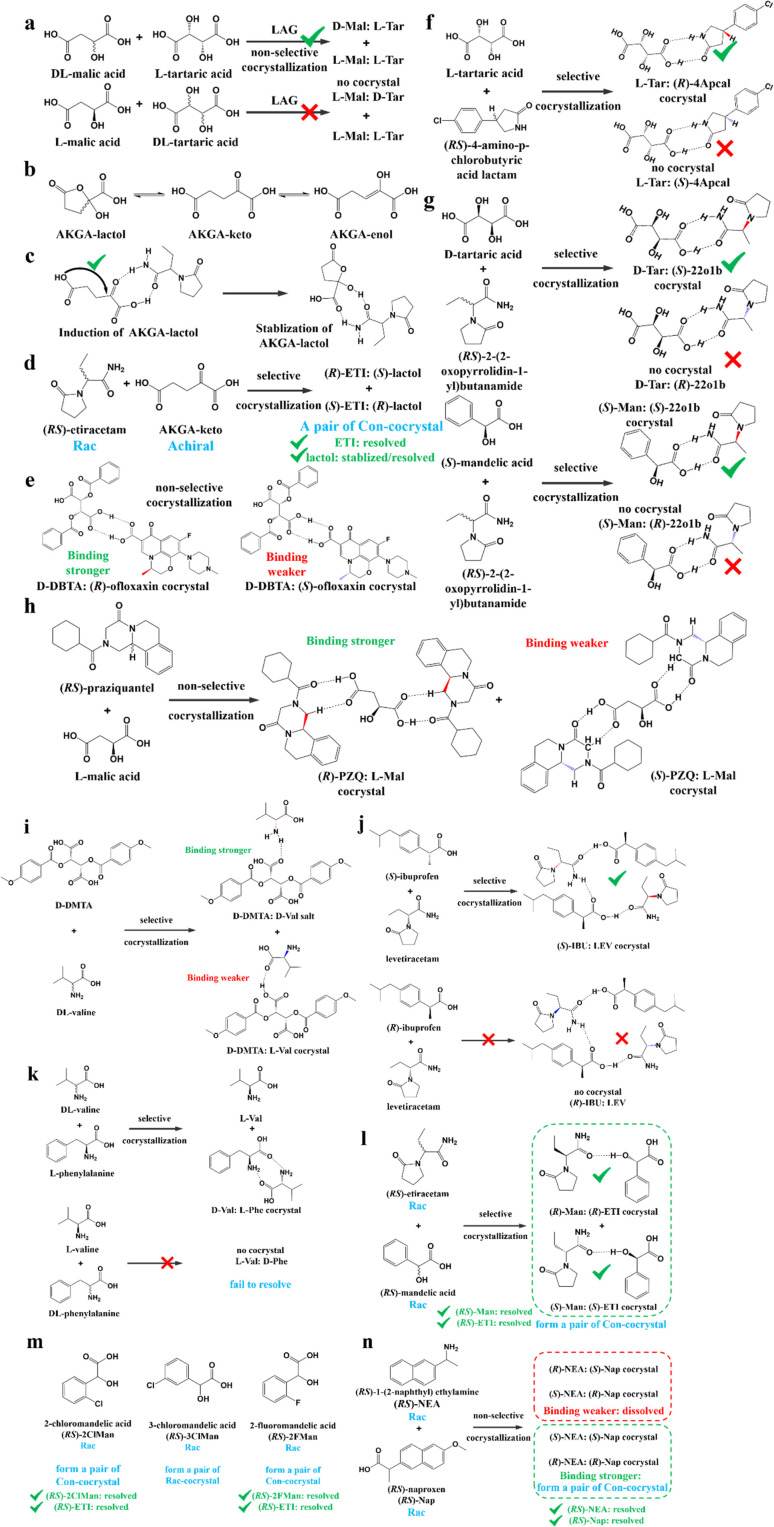
Typical resolution cases based on selective or non-selective cocrystallization. (a) Successful resolution of dl-Mal using chiral Tar by forming non-selective diastereomeric cocrystals, but not *vice versa*. (b) AKGA tautomers, (c) cocrystal-induced selective stabilization of the lactol form, and subsequent reciprocal resolution of (*RS*)-ETI and AKGA by forming enantiospecific cocrystals. (e) Non-selective diastereomeric cocrystals formed between d-DBTA and ofloxacin enantiomers to achieve resolution. (f) Enantiospecific cocrystal formation between l-Tar and (*R*)-4Apcal to achieve resolution. (g) Enantiospecific cocrystal formation between (*S*)-22o1b and d-Tar or (*S*)-Man to achieve resolution. (h) Non-selective diastereomeric cocrystals formed between l-Mal and PZQ enantiomers to achieve resolution. (i) Selective cocrystal and salt formation between d-DMTA and valine enantiomers to achieve resolution. (j) Enantiospecific cocrystal formation between (*S*)-IBU and LEV to achieve resolution. (k) Successful resolution of valine enantiomers using chiral Phe by forming enantiospecific cocrystal, but not *vice versa*. (l) and (m) Simultaneous reciprocal resolution of (*RS*)-ETI and (*RS*)-Man, (*RS*)-ETI and (*RS*)-2ClMan, or (*RS*)-ETI and (*RS*)-2FMan *via* enantiospecific cocrystal formation. (n) Simultaneous reciprocal resolution of (*RS*)-NEA and (*RS*)-Nap under proper crystallization conditions, despite the formation of four non-selective diastereomeric cocrystals.

For example, diastereomeric cocrystals can be formed *via* liquid-assisted grinding (LAG) between l-tartaric acid (Tar) and dl-malic acid (Mal). However, l-Mal fails to discriminate Tar enantiomers in a racemate (Fig. 12a). Therefore, enantiopure Tar can resolve Mal enantiomers, while the reverse case is not feasible. This may be because Tar possesses a higher melting point and lower lattice energy than the two diastereomeric cocrystals.^229^ George *et al.* synthesized the metastable (Fig. 12b) lactol tautomer of α-ketovaleric acid (AKGA) by forming diastereomeric cocrystals between its stable achiral ketone form, AKGA-keto, and (*RS*)-etiracetam (ETI).^230^ Interestingly, the stable form of AKGA could be converted from the keto and enol forms into the lactol form in the presence of ETI molecules. The construction of the intramolecular interactions allows an *R*_2_^2^ (8) motif and overcomes the metastability of the lactol form (Fig. 12c). The ETI-AKGA-keto cocrystal (Rac) was inclined to transform into a stable ETI-AKGA-lactol cocrystal (Con) in solution (Fig. 12d). Therefore, both the racemic compound (*RS*)-ETI and achiral AKGA could be resolved to their respective stable enantiomers. In 2018, He *et al.* reported the formation of non-selective cocrystals between d-DBTA and chiral ofloxacins and achieved ofloxacins with 82.3% ee_*R*_ and 81.8% ee_*S*_ (Fig. 12e).^231^ Similarly, l-Mal could form a pair of diastereomeric cocrystals with (*RS*)-praziquantel ((*RS*)-PZQ) (Fig. 12h). In this system, (*R*)-PZQ·L-Mal possessed significantly lower solubility than (*S*)-PZQ·l-Mal in ethyl acetate.^232^ Consequently, (*R*)-PZQ·L-Mal underwent direct crystallization and (*R*)-PZQ with 99.3% ee could be subsequently liberated from the cocrystal. In 2022, (+)-di-*p*-anisoyl-d-tartaric acid (d-DMTA) was employed to separate dl-valine (dl-Val). Interestingly, the two diastereomers consisted of a metastable cocrystal and a stable salt (Fig. 12i). This is because the supramolecular synthon of d-DMTA:d-Val salt possessed higher intrinsic stability and a more negative solvation free energy than that of the d-DMTA:l-Val cocrystal. Therefore, d-Val with a higher profit margin could be obtained (96.6% ee and 40.68% yield).^233^

Alternatively, enantiospecific cocrystals (iv) are more innovative because they ideally involve only one new solid cocrystal phase. Consequently, this contributes to a less complex phase diagram and a larger solubility difference between the cocrystal and single enantiomer for direct crystallization. The earliest example is the cocrystal involving l-Tar and (*R*)-4-amino-*p*-chlorobutyric acid lactam, leaving the (*S*)-isomer in the mother liquor (Fig. 12f).^234^ Further computational crystal structure predictions for three enantiospecific and diastereomeric cocrystals indicated that the appearance of the *R*_2_^2^ (8) dimer may contribute to the formation of enantioselective cocrystals.^235^ Leyssens *et al.* confirmed this principle in two other enantiospecific systems, where both (*S*)-Man and d-Tar enantiospecifically cocrystallized with (*S*)-2-(2-oxopyrrolidin-1-yl)butanamide (Fig. 12g).^236^ Therefore, the potential strategy for preparing stereospecific cocrystals seems to be finding CCF with the least probability of forming alternative motifs except for *R*_2_^2^ (8) dimers. Moreover, a novel strategy achieving the synergy of chiral resolution and dual-drug preparation was reported *via* enantiospecific cocrystallization between levetiracetam (LEV) and ibuprofen (IBU) (Fig. 12j).^237^ However, only a partial Ss (ee_*S*_ = 94%) could be obtained after the liberation of the cocrystal. Thus, to address this problem, NaCl was introduced to form an IBU·NaCl salt to narrow the fan-shaped area of the Rac (FE_1_r in Fig. 7b), thereby producing (*S*)-IBU·NaCl with 99% ee from the previous crude Ss product. Hence, the enantiospecific cocrystal system may not necessarily eliminate the formation of Ss and the contamination pathway of the undesired (*R*)-enantiomer still remains ambiguous. In 2021, enantiopure valine and phenylalanine were mutually employed as CCF for each other.^238^ A non-interchangeable resolution phenomenon similar to that in Fig. 12a was observed although the cocrystal system exhibited enantioselectivity (Fig. 12k). This is because the thermodynamic stability of the enantiospecific cocrystal is higher than that of dl-valine, while lower than dl-phenylalanine.

**Fig. 13:**
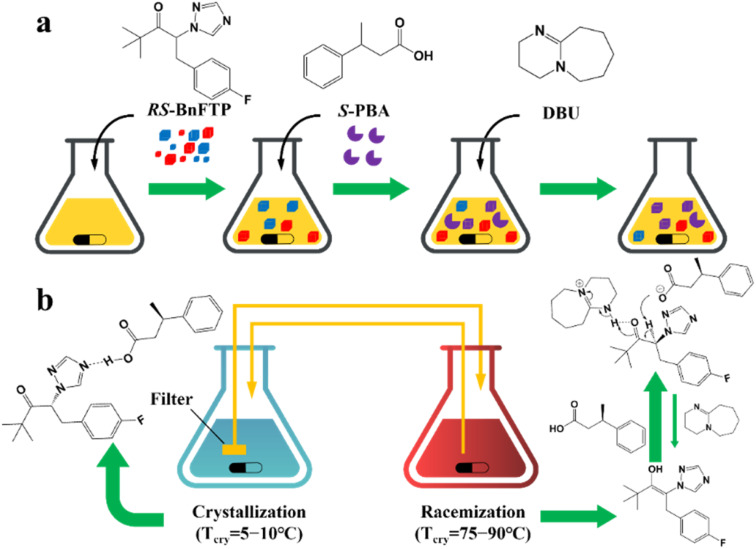
(a) Schematic representation of the CoISD process and (b) the CoISD mechanism that occurred in a two-vessel setup with the cocrystal in suspension.^55,239^*RS*-BnFTP, PBA and DBU represent the racemic mixture, chiral coformer and racemizing agent, respectively.

Additionally, coupling strategies can be used for cocrystal-based separation processes. For instance, Leyssens *et al.* devised a cocrystallization-induced spontaneous deracemization (CoISD) set-up by integrating racemization with CBR (Fig. 13). After the direct crystallization of the stable *S*-BnFTP:*S*-PBA cocrystal, the more soluble *R*-BnFTP:*S*-PBA complex was left in the mother liquor and the *R*-BnFTP molecules continuously racemized to their counterpart under the catalysis of DBU. Given that DBU is incapable of racemizing *S*-PBA, *R*-BnFTP:*S*-PBA was converted to *S*-BnFTP:*S*-PBA in this coupled setup.^239^ After 4 days of batch operation *via* liquid phase circulation, enantiomers with over 99.9% ee and 50.7% yield were prepared. A further racemization kinetics study suggested that the presence of acids other than DBU can shift the racemization reaction from first-order kinetics to a more complex situation.^240^ Consequently, it is necessary to resolve the kinetics of both crystallization and racemization by setting them at distinct temperatures despite the current lack of systematic guiding principles. Also, the catalyst should not racemize the CCF. Otherwise, the *R*-BnFTP:*R*-PBA cocrystal would crystallize and contaminate the solid phase. Notably, a minor limitation of this study is that the racemate used is Con, while it appears that extending this strategy to Rac will be a more promising approach.

Recently, inspired by the previously reported enantiospecific *S*-mandelic acid:*S*-etiracetam cocrystal (*S*-Man:*S*-ETI) system,^241,242^ Zhou *et al.* reported a cost-effective and key reciprocal resolution phenomenon. Unexpectedly, equimolar *RS*-Man and *RS*-ETI could be innovatively used as their reciprocal chiral coformers, thereby transforming the physical mixture of two Rac racemates into a new Con composed of two equimolar cocrystals, *R*-Man·*R*-ETI and *S*-Man·*S*-ETI (Fig. 12l). Therefore, two Racs can be simultaneously resolved in a PC process by alternately adding seeds of two cocrystals (MM′NN′ trajectory in Fig. 7a) and the liberation of cocrystals. The ee values of Man and ETI reached about ≈95% and >98%, respectively.^243^ Moreover, in 2023, the same group further reported that *RS*-ETI:*RS*-2ClMan and *RS*-ETI:*RS*-2FMan crystallized as Con-cocrystals, while *RS*-ETI:*RS*-3ClMan served as an Rac-cocrystal due to the lack of intramolecular halogen bonds of C–Cl⋯O (Fig. 12m).^244^ This indicates that *RS*-ETI:*RS*-2FMan or *RS*-ETI:*RS*-2ClMan can also undergo simultaneous resolution of two Racs *via* PC. Inspired by this strategy, in the same year, Hao *et al.* reported another PC-CBR-coupled strategy to simultaneously resolve two Racs, (*RS*)-naproxen and (*RS*)-1-(2-naphthyl) ethylamine (Fig. 12n).^245^ Hence, previously reported chiral cocrystals, in particular those possessing higher thermodynamic stability than their pristine racemates, may provide upgraded versions for developing more cases of “reciprocal resolution”.

Although CBR can provide the advantages of an eco-friendly process, enantiospecific separation, and the potential for unexpected surprise, it presents a challenge when defining the boundary between “cocrystals” and “salts” during coformer design. This may arise from the lack of clarity in the cocrystal formation mechanism and efficient prediction method. At present, achiral coformers contribute to PC and PE processes by forming novel Racs or Cons, respectively, while chiral coformers bring both thermodynamic and kinetic differences between two antipodes, thus contributing to direct crystallization. However, the existing CCF scope is confined to excellent hydrogen-bonding capabilities and steric hindrance in compounds such as Tar, Man, Mal, and their derivatives. New developments in CCFs should make full use of other non-covalent interactions (*e.g.*, halogen bonds and π–π interactions), the CSD database, and computational chemistry simulation. In addition, it is cumbersome to construct quaternary phase diagrams for both CBR and CCR because multiple solid phases may coexist in the system. Hence, combining multiple online characterization methods, such as HPLC, PXRD, UV, CD, and FTIR,^246^ or constructing quasi-quaternary phase diagrams^238^ may half the work with double the results.

#### 4.4.4 Dutch resolution

DR was first proposed by Vries in 1998 and was officially named in 2000.^247^ The main difference between DR and CCR/CBR is that a group of structurally related resolving agents is used in the DR instead of a single resolving agent. For example, Kaptein *et al.* realized the enantioselective nucleation and crystal growth inhibition toward the undesired diastereomer with which the DR family members are liable to form Ss.^29^ In addition, achiral molecules can also be added to the DR resolving agent to inhibit the crystallization kinetics of the distomer. Leeman *et al.* achieved an enantiopurity of 95% by adding bifunctional achiral nucleation inhibitors to the family agent.^56^ However, the challenge in achieving DR lies in the efficient self-assembly of multiple molecules in the crystal phase. For instance, a Dutch agent composed of a mixture of two chiral host diols failed to resolve 2-butylamine because two types of pockets were constructed in the host–guest complex. Specifically, one type of pocket enantioselectively incorporated the *R*-enantiomer due to the good size matching, while the other loosely stacked pocket exhibited indiscriminate adsorption for two enantiomers. This failure can be ascribed to the difficulty in increasing the proportion of the first pocket through molecular self-assembly.^248^

**Fig. 14:**
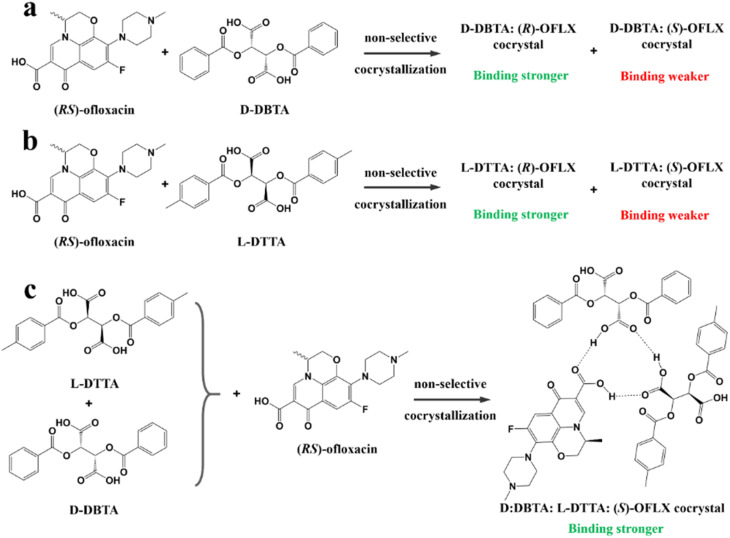
(a) Non-selective diastereomeric cocrystals formed between d-DBTA and ofloxacin enantiomers to achieve resolution. (b) Non-selective diastereomeric cocrystals formed between l-DTTA and ofloxacin enantiomers to achieve resolution. (c) Chiral switching phenomenon observed in (*RS*)-ofloxacin resolution, where l-DTTA and d-DBTA serve as Dutch resolving agents.^30^

Additionally, the chiral switch of the preferred enantiomer is a fascinating phenomenon in Dr Kodama *et al.* synthesized a series of binary cocrystal-based resolving agents by combining l-DBTA and various achiral diamines. The as-prepared resolving agents resulted in dimensionally different hydrogen bonding networks with secondary alcohols by altering the structure of the achiral diamine. When the achiral diamine changed from 4,4′-bipyridine to 2-phenylimidazole, the enantiospecific recognition of (±)-benzylic alcohols changed from *S*- to *R*-isomer.^249^ Besides, 1,1′-bi-2-naphthol derivatives, which possess skewed conformations of aromatic rings such as DBTA, displayed similar influences on the obstruction of dense packing and the promotion of enantioselective inclusion. Notably, the most intriguing finding is that the resolution outcomes of ofloxacin enantiomers using a DR agent composed of two Tar derivatives (DBTA and DTTA) are contrary to that observed in CBR.^30^ Specifically, l-DTTA or d-DBTA formed a stable cocrystal with *R*-ofloxacin in CBR, while the DR agent combining l-DTTA and d-DBTA led to a chirality switch from *R*- to *S*-ofloxacin. This is because three O–H⋯O interactions are involved to form a stable supramolecular synthon consisting of *S*-ofloxacin, l-DTTA and d-DBTA molecules (Fig. 14). Consequently, ee values of 41.15% for *S*-ofloxacin and 27.10% for *R*-ofloxacin could be obtained within 30 min using a 1 : 1 molar ratio of “DBTA + DTTA” under the optimized conditions.

Compared with CCR and CBR, DR displays adaptability to oil/amorphous-forming systems owing to the structural similarity and stereochemical uniformity of their family members. To date, these exotic phenomena have been attributed to the formation of solid solutions between the salts of family members or a kinetic process in which a particular resolving agent hinders the nucleation of high-solubility diastereomers.^31,32^ Furthermore, there are similarities between the DR agents and tailor made-additives in terms of inhibiting the nucleation of distomer, although additives do not appear in the crystal structure of the target enantiomer unless they form a solid solution. Accordingly, further elucidation of this issue may guide the design of DR agents.

## 5. Deracemization process coupling racemization and crystallization

Deracemization is a promising approach to ultimately convert a racemate to a single-handed product with the addition of a racemizing agent. Hence, the concentration difference between two enantiomers serves as the most significant driving force to achieve deracemization, which combines racemization and another enantioseparation method. At present, crystallization is the most economical approach to achieve deracemization given that it can naturally achieve the “product removal process” for the racemizing reaction by enriching the excess enantiomer in the solid phase. These techniques include crystallization-induced diastereomer transformation (CIDT),^38^ second-order asymmetric transformation (SOAT),^250^ dynamic preferential crystallization (DPC),^251^ temperature cycling-induced deracemization (TCID), and Viedma ripening (VR). Among them, DPC and CIDT suit the deracemization of conglomerates and diastereomers, respectively, while VR, TCID and SOAT are available to both systems. Notably, VR and TCID can be considered as state-of-art versions because they ingeniously utilize simple physical conditions (glass beads, ultrasound, and temperature cycles) to induce the enantioselective dissolution of chiral crystals, providing a continuous driving force for the racemization process.

### 5.1 Viedma ripening

VR refers to the groundbreaking discovery in 2005 when Viedma reported that achiral NaClO_3_ crystals (Con) could be randomly converted to either d or l-crystals during the crystallization process in a stirred solution containing glass beads.^84^ This phenomenon can be attributed to the size-dependent solubility differences induced by the attrition of the glass beads, where smaller crystals dissolve, while larger ones continue to grow. For instance, if d-crystals become dominant in the solid phase beforehand, they will absorb more d-NaClO_3_ molecules on their surface for crystal growth. Due to the inherent rapid racemization kinetics between the two enantiomers in the solution, the small l-crystals will dissolve and contribute to the growth of d-crystals through racemization. Further attrition of the larger d-NaClO_3_ crystals will provide more growth sites for d-molecules due to the secondary nucleation. Ultimately, infinitesimal self-catalytic cycles in VR can lead to a racemic mother liquor and enantiopure solid phase by using glass beads grinding at low supersaturation.

**Fig. 15:**
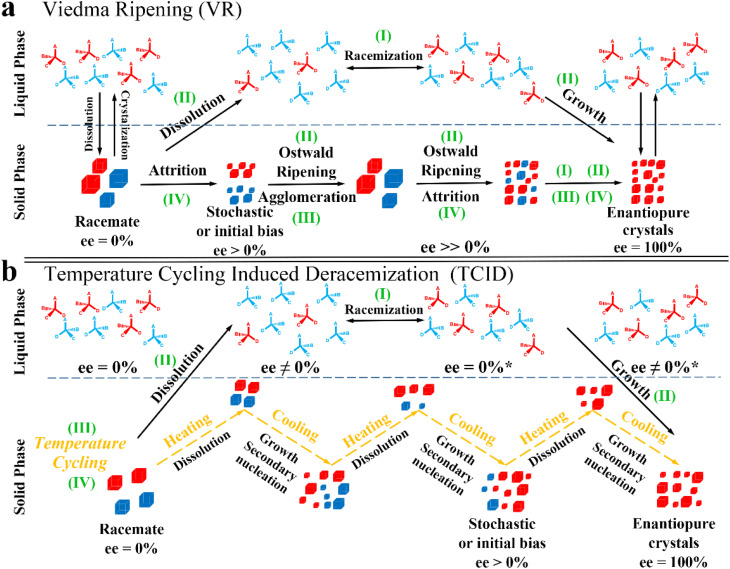
Schematic representation (a) VR and (b) TCID mechanisms of a Con.^259^ (I−III) Represent racemization, Ostwald ripening and agglomeration/cluster incorporation, respectively. (IV) Represents attrition (for VR) or size/solubility differences resulting from temperature fluctuations (for TCID). Red and blue molecules represent d- and l-enantiomer, respectively.

With the emergence of studies on different initial conditions (ee_0_, the amount of racemizing agent and glass beads) and chiral molecules (NaBrO_3_, NaIO_3_, amino acids and their derivatives),^84^ VR can be summarized as a convergence of many circumstances (Fig. 15a), as follows: (I) appropriate liquid-phase racemization conditions compatible to crystallization. If the substrate cannot undergo spontaneous racemization, organic acids or bases should be added as a resolving agent catalyst.^252–254^ (II) Ostwald ripening. The growth of large crystals is at the expense of tiny ones, resulting in changes in particle size distribution over time.^255,256^ (III) Agglomeration between homochiral pre-nucleation aggregates, which ensures the crystal growth of enantiopure crystals. Consequently, it is significant that the system should be a Con or a pair of diastereomers. (IV) Ultrasound and/or glass bead-induced attrition, and the addition of enantiopure seeds if necessary. Ultrasound grinding produces small crystal pieces with a fast racemization rate, while glass bead grinding generates large fragments with a relatively slower racemization rate.^257^ This is because pure ultrasound can significantly promote the secondary nucleation in the initial period in relation to glass beads, while continuous ultrasound may homogenize the crystal size, decrease the size-dependent solubility gradients, and diminish the speed of kinetic-dependent crystal growth. Thus, it is reasonable to apply both beads and ultrasonic grinding to speed up VR.^257^ However, a more realistic scenario is that enantiomer E^+^, with crystals formed beforehand in larger sizes, tends to remain in the solid phase due to its lower solubility, while enantiomer E^−^, with a larger number of crystals in the suspension, tends to be replenished in the liquid phase *via* the racemizing reaction due to its faster solute consumption rate, thereby causing the dissolution of larger crystals of E^+^. This indicates that the mechanism of crystallization-based deracemization is a complex trade-off between thermodynamics and kinetics, which was systematically reviewed by Buhse *et al.*^258^

In recent years, VR has evolved beyond the deracemization of Cons and has generated multiple innovative coupling variants that are applicable to complex racemate systems. In 2022, VR was integrated with CCR to deracemize dl-PGA by DBU after forming salts with the resolving agent dl-NAT. This system included five salt complexes whose thermodynamic stability followed the order of d-PGA:l-NAT (dl) = l-PGA:d-NAT (dl) < d-PGA:d-NAT (dd) = l-PGA:l-NAT (ll) < dl-PGA:dl-NAT (dldl). Hence, the preparation of useful dl salts with high de values faces the challenge of being epimerized to ll or simultaneously precipitating with other stable salts. By using PXRD and DSC monitoring, they found that different solid phases existed in different periods. Specifically, VR only took place within 88 h, during which ld was continuously converted to dl, allowing the dl salt to be the only solid phase for 113 h. During 113–240 h, metastable dl was dissolved and transformed into stable ll, and the nucleation of dd and ll was initiated. Ultimately, deracemization longer than 240 h resulted in the most stable phase of dldl. Hence, target dl salt can only be harvested in the “window period” (88–113 h) and the deracemization must be done within 88 h.^260^

**Fig. 16:**
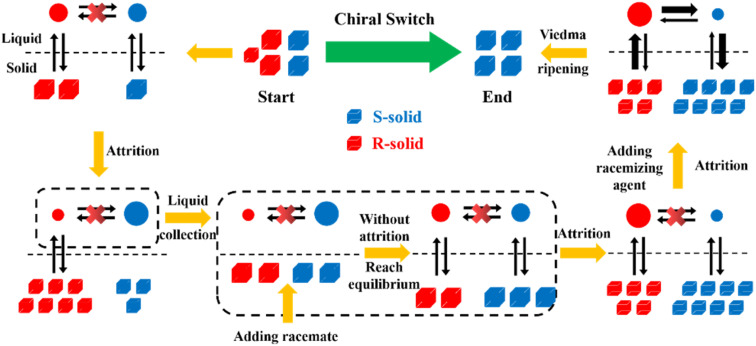
Stepwise schematic illustration of the “chiral switch” approach.^261^

In general, the racemizing agent and grinding serve as kinetic strategies to maintain the system in a non-equilibrium state throughout the VR process, resulting in the deracemization of the raw material into the initially somewhat excess enantiomer. However, in 2019, Noorduin and coworkers designed a “chiral switch” process, which could counterintuitively deracemize the somewhat *R*-enriched solid phase to *S*-crystals (Fig. 16).^261^ Specifically, the starting material first generated an *R*-enriched solid phase and *S*-enriched liquid phase due to the racemization-free attrition conditions. The as-obtained liquid phase was collected and further mixed with racemic conglomerate crystals in the absence of attrition to reach the equilibrium state, transferring the excess *S*-enantiomer in the filtrate to the solid phase. Accordingly, the addition of racemizing agent and attrition can produce enantiopure *S*-crystals because of the sufficient concentration of the *R*-enantiomer in the liquid phase and the affluent total crystal surface of the *S*-enantiomer in the solid phase. It should be noted that this “chiral switch” can serve as a novel strategy to deracemize non-racemic starting materials into products of the initially non-prevalent enantiomer. Furthermore, as described in Section 4.1, many racemates can crystallize as either stable Con or metastable Rac under different crystallization conditions. Engwerda *et al.* found that when using metastable Rac as the feed and introducing the racemizing agent after a period of grinding, a shorter grinding time counterintuitively accelerated the VR process.^87^ This is because long-term grinding generates more stable fine crystals of Con with uniform size distribution, leading to an additional, time-consuming dissolution step of enantiopure crystals during deracemization. By comparison, the metastable Rac crystals exhibit higher dissolution efficiency during short-term grinding, resulting in a fast deracemization. This accelerated VR variant is particularly available for racemates whose Con and Rac possess relatively close thermodynamic stability.

**Fig. 17:**
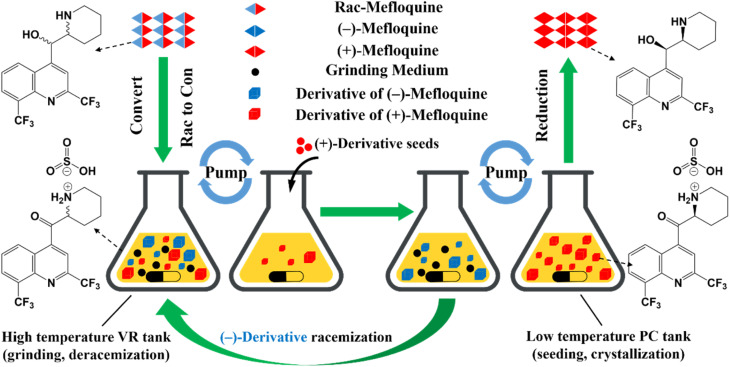
Experimental chemical derivatization-VR-PC coupled setup used for the deracemization of mefloquine.^262^ Blue arrows: crystal-free liquid exchange through tubes.

**Fig. 18:**
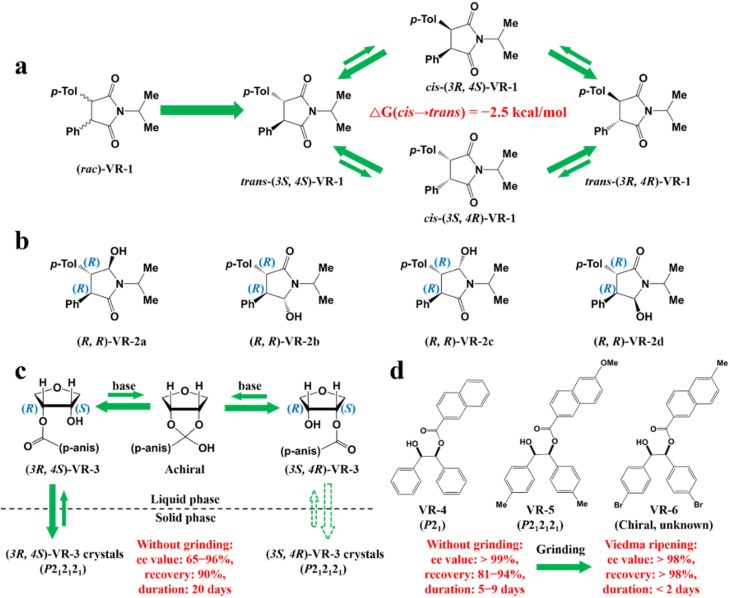
(a) Epimerization and/or racemization of (*rac*)-VR-1 take place *via* reversible deprotonation of the stereocenters using DBU as the catalyst. (b) Additives, VR-2a to VR-2d, for directing the outcome of the VR experiments on compound (*rac*)-VR-1. In all cases, all four (in this case (*R*, *R*)-based) additives were used together.^263^ Chiral symmetry-breaking mechanism and outcomes of (c) monoacylated sugar VR-3 by enantioselective crystallization and racemization and (d) monoacylated 1,2-diaryl-1,2-ethanediols VR-4 to VR-6 by Viedma ripening.^264^

In addition, it is highly valuable to expand the applicability of VR to complex Rac/Ss systems with more than one chiral center that are challenging to undergo racemization, even if they do not meet prerequisites (I) and (III) of VR. For example, Anthonius H. J. Engwerda *et al.* devised a VR process for a Rac, mefloquine (Mef), which possesses two difficult-to-racemize chiral centers (Fig. 17).^262^ Mefloquine was ingeniously screened and transformed to its racemic derivative, sulfonate of ketone, which has only one racemizable chiral center and crystallizes as a Con. Moreover, (±)-Mef derivative crystals can be transformed to (+)-(*S*)-Mef derivative crystals in a VR-PC coupling setup with crystal-free liquid-phase exchange. Specifically, the addition of glass beads to the VR tank could continuously deracemize the (±)-(*RS*)-derivative to (−)-(*R*)-crystals, while maintaining a racemic state in the liquid phase for a further (+)-(*S*)-seed-induced PC process. It is important to note that high and low temperatures are beneficial to the kinetics of racemization and crystallization, respectively. Faster racemization kinetics, compared to crystallization, is crucial to ensure that the solution maintains a “racemic state”, which can consecutively provide the driving force for PC process. Ultimately, the as-prepared (+)-(*S*)-derivative crystals could undergo a Luche reduction to obtain the target (+)-(11*S*,12*R*)-mefloquine (94% ee and 83% yield), with acceptable chiral impurities involving 3.5% (11*R*,12*R*)-mefloquine and 2.5% of (−)-(11*S*,12*R*)-mefloquine. Meanwhile, (−)-(*R*)-derivative crystals in VR vessel could be recycled as the feed after undergoing racemization. Therefore, compared to individual chiral separation methods, the coupling strategy involving VR, PC, and chemical derivatization is of great significance to transform complex racemic mixtures into an optically pure chiral compound. In another example, VR was coupled with reverse PC for the deracemization of a Con, which possesses two stereocenters and four stereoisomers (Fig. 18a).^263^ With the addition of the racemizing agent DBU, chiral centers of *cis* (3*S*,4*R*)- and (3*R*,4*S*)-VR-1 consisting of the initial Con crystals could be dynamically epimerized to stable *trans* (3*R*,4*R*)- and (3*S*,4*S*)-VR-1 due to the negative free energy difference. Hence, VR can take place between two *trans* enantiomers according to the random chiral evolution. Moreover, four additives obtained through facile epimerization, from *trans*, (*S*, *R*) and (*R*, *S*), to *cis*, (*R*, *R*) and (*S*, *S*), under the catalysis of DBU, together with the generation of two *trans*-VR-1 enantiomers in the same VR container, collectively acted to induce VR towards specific chiral preference. According to the “rule” (Section 4.2.3), the adopted (*R*, *R*)-related additives inhibited the crystallization kinetics of (3*R*,4*R*)-molecules, while promoting the PC of enantiopure (3*S*,4*S*)-crystals, reducing the total VR time from 100 h to 2.5 h.^263^ It is evident that the coupling of VR with additive- or seed-based (reverse) PC can effectively disrupt the stochastical evolution during chiral amplification by introducing an initial chiral preference, enabling fast and efficient deracemization of the starting material to the desired enantiomer.

Furthermore, studies have shown that chiral compounds based on the same deracemization mechanism can be accelerated by VR. In 2022, Sanada *et al.* investigated a representative Con system, the monoacylated sugar O-anisoyl anhydroerythritol (VR-3), which underwent intramolecular acyl transfer under the racemizing effect of DBU. Notably, it took 20 days for a dynamic crystallization process to achieve deracemization (Fig. 18c). By comparison, using VR for Cons, monoacylated 1,2-diaryl-1,2-ethanediols (VR-4–VR-6), which underwent the same deracemization mechanism with VR-3, could significantly reduce the deracemization time by 3–7 days (Fig. 18d).^264^ Therefore, VR has promising prospect to present the time-consuming chiral evolution processes of large biomolecules in nature as a “fast-forward mode”, rendering it convenient to discuss the origin of chirality or the reasons why d-sugars and l-amino acids are dominant in living organisms.

Although VR has shown excellent coupling potential, fast deracemization speed and controllable crystal size distribution, limited research investigated the impact of the material, size, and amount of grinding medium on the outcomes. Additionally, the removal of the grinding medium may cause the loss of some enantiopure products and a lower overall yield. By comparison, TCID can avoid the removal of the attrition medium and serve as a major complement to VR.

### 5.2 Temperature cycle-induced deracemization

Another practical pathway to induce mirror symmetry breaking is TCID, which can generate size-dependent solubility by substituting temperature cycles for the grinding medium.^41,265^ The temperature-cycling program usually consists of a heating stage, high-temperature isothermal stage, cooling stage and low-temperature isothermal stage, sequentially. Therefore, its mechanism involves racemization, Ostwald ripening and agglomeration under lower supersaturation (Fig. 15b). During temperature fluctuations, the periodic dissolution-crystallization events will induce stochastical nucleation of both enantiomers and lead to an initial bias in the solid phase, where one enantiomer exceeds the other in terms of the number or size of crystals. This allows the dominant d-crystals to be partially retained in the subsequent dissolution process, while the l-enantiomer dissolves from the crystals and is rapidly racemized to its counterpart for the growth of d-crystals (Fig. 15b). Therefore, temperature cycling can continuously amplify the slight enantiomeric imbalance in the solid phase caused by random nucleation, thereby converting dl-crystals into d-crystals larger than that of VR due to the absence of attrition. Normally, the disproportionate Ostwald ripening of two enantiopure crystals and temperature-dependent racemization kinetics are essential factors for TCID, while agglomeration and fragmentation seem less important although seeding, initial ee_0_ ≠ 0, and slight attrition can accelerate the process.^266^ By comparison, vigorous grinding intensity sufficient to induce VR may switch the evolution itinerary and be detrimental to a high ee value.^267^

**Fig. 19:**
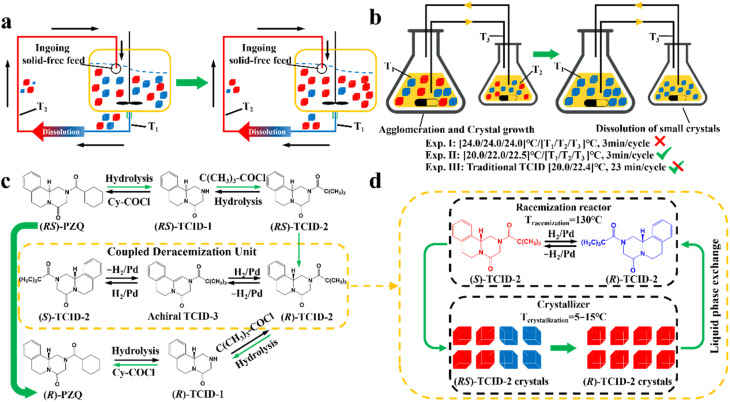
(a) Schematic representation of the total spontaneous resolution setup. *T*_1_: suspension temperature and *T*_2_: dissolution temperature (*T*_1_ < *T*_2_).^268^ (b) Experimental setup used for TCID *via* coupled mixed-suspension vessels. *T*_1_: temperature of the cool vessel; *T*_2_: temperature of the hot vessel and *T*_3_: temperature of the tube.^86^ Illustration of (c) global (*RS*)-PZQ resolution procedures and (d) chemical derivatization-TCID-PC-coupled system for deracemization.^91^

In recent years, numerous coupled TCID variants have been reported to enhance the productivity and efficiency of TCID rather than simply modifying and optimizing the operation variables such as temperature fluctuation range,^269–271^ cooling rate,^41,269,270^ initial ee value,^269,270,272^ introduction of additives,^273^ concentration of the catalyst/substrate,^269^ racemization rate,^271^ amount of solids dissolved in each heating–cooling cycle,^41,269,274^ and volume of the system.^270,274^ For example, Steendam *et al.* presented a homogenization integrated TCID device, where a whisk was employed to homogenize large agglomerates and provide seeds, but it was incapable of inducing VR (Fig. 19a). Notably, this device replaced the complex temperature cycling program with slurry recycling to achieve the partial dissolution of *S*-crystals, thus spatially disengaging the dissolution and crystal growth into the high-temperature tube and low-temperature crystallizer, respectively. Ultimately, NaBrO_3_ (Con) could be deracemized to a single enantiomer with >98% ee.^268^

Another variant concerning two-pot TCID with liquid-phase coupling shows considerable merits such as fast deracemization kinetics,^41^ ease of temperature control,^275^ good scaling potential, and a short temperature cycle period.^86^ In this setup, the temperature fluctuation procedure is partially replaced by a circulating operation of the suspension between the high-temperature dissolution tank and the low-temperature crystallization tank (Fig. 19b). Hence, the absence of temperature differences would result in the absence of the driving force of partial dissolution and crystal growth, and subsequent inability to achieve chiral amplification (Exp I). By comparison, temperature differences among the hot vessel, cold vessel, and tube, and a shorter temperature cycle period (3 min per cycle) could achieve the total deracemization of a zero initial ee_0_ Con within 32 h (Exp II). Notably, traditional TCID with a longer period (23 min per cycle) still consumed an additional 8 h compared to the two-pot operation (Exp II), albeit with an initial ee_0_ of 13% to accelerate the process (Exp III).^86^ This may be because the two-pot setup can provide more infinitesimal temperature fluctuations. After the bulky dissolution and racemization of the distomer in the hot vessel, the remaining undissolved eutomer crystals can further grow in the low-temperature crystallizer with a longer residence time, which allows for the conversion to the single-handedness product.

In addition, these two-pot strategies can be further coupled with cocrystal/derivatization to orient TCID to Racs that cannot satisfy conditions (I) and (III) (Fig. 15b). For example, in 2021, to achieve the deracemization of an Rac, praziquantel (PZQ), which fails to undergo liquid-phase racemization, Valenti and coworkers screened potential Cons from their derivatives *via* hydrolysis and acylation reactions (Fig. 19c).^91^ Fortunately, PXRD, SHG and DSC characterization confirmed that the as-synthesized (*RS*)-TCID-2 serves as a Con and condition (III) was satisfied. Alternatively, the undesired (*S*)-TCID-2 could be deracemized to (*R*)-TCID-2*via* Pb/H_2_-catalyzed hydrogenation–dehydrogenation reactions including an achiral intermediate, TCID-3, thereby satisfying prerequisite (I). Hence, the deracemization of (*RS*)-TCID-2 could be achieved in a coupled setup involving a Pd/H_2_-packed reactor and a crystallizer with temperature fluctuations (Fig. 19d). Compared to one-pot TCID, the crystallization and racemization were separated in their respective devices *via* the crystal-free liquid phase circulation. Ultimately, (*R*)-TCID-2 could be afforded with 98% ee and 32% yield within 18 h, allowing the subsequent production of the target (*R*)-PZQ.

TCID is a complementary method to VR, which can provide enantiopure products with larger size and higher yield and has promising prospect to develop more liquid-phase coupling setups for total deracemization. However, it may be strenuous to determine the optimal conditions of TCID because the temperature-controlling program almost doubles the variables involved.^41,269,270^ Consequently, it is challenging to develop PBMs to describe this process.

### 5.3 Other novel deracemization approaches

At present, there are few publications discussing the integration process of VR and TCID despite their respective availability for achieving high enantiopurity. In 2022, Belletti and coworkers studied the combination effect of VR and TCID on a Con, (2-methylbenzylidene)-phenylglycine amide.^88^ They believe that there is a critical value for the temperature cycling range (Δ*T*) of TCID. Specifically, Δ*T*, which is higher than that value, corresponds to TCID-dominant deracemization, where the introduction of grinding is detrimental to deracemization. By comparison, a lower Δ*T* represents VR-dominant processes, where the deracemization can be accelerated as a function of grinding speed until a critical value of the grinding intensity is reached. Hence, this concept can be applied to prepare fine or bulky crystals with high enantiopurity according to the relative strengths of the TCID program and grinding mode.

However, numerous adjustable variables exist in the deracemization process such as initial conditions (initial ee_0_), operation mode (liquid coupling, grinding mode, T-cycling mode, *etc.*), seeds (size distribution, ee, load, *etc.*), and kinetic parameters (crystal growth, nucleation, and racemization), and the significance of these parameters necessarily alters with Cons. One method to tackle this issue is to establish generic shortcut models containing only simplified key parameters related to nucleation, crystal growth and racemization. For instance, Seidel-Morgenstern and coworkers applied their previous PC shortcut models to the deracemization of Cons.^60^ The shortcut models were employed to predict outcomes of racemase-based deracemization for dl-asparagine monohydrate. As guided by the model, the productivity of the target enantiomer witnessed an increase of 45% within just one-third of the duration despite a decrease of 10% yield. Hence, the development of shortcut models can determine optimal operating conditions without the need for precise kinetic parameters. Another approach is to establish generalized models to determine the significance of the operational parameters and to orient the model to extensive racemates. For instance, in 2023, Kovács and coworkers constructed the Random Forest- and XGBoost-based machine learning models according to multitudes of previous VR and TCID cases.^276^ The resulting regression model allows for the importance ranking of the eight most widely discussed operational variables based on their weight factors. Specifically, the racemization rate has the most prominent effect on the final ee values of both TCID and VR, and the relative size and ee of the seeds serve as the second important factor for VR and TCID, respectively. However, it seems less effective to increase the final ee by modifying the growth rate during deracemization and the nucleation rate is more influential on VR than TCID. Appealingly, the nucleation and growth kinetic-excluded model still retained its accuracy for predicting the order of significance. This is because machine learning models are established from optimal process operations, which have already connected “good deracemization outcomes” with “good kinetic conditions”, and thus the absence of these two difficult-to-determine parameters will not significantly reduce the accuracy of this generalized predictive model.

In 2023, Noorduin and coworkers proposed a counterintuitive conclusion on deracemization influenced solely by crystal growth, almost without nucleation and attrition, by adding crystal seeds under slight shaking conditions.^277^ By modifying the concentration of the resolving agent, they found that relatively faster or slower racemization relative to crystallization led to ee values of the newly grown crystal layer (ee_Δ_) higher than or almost equal with that of seeds (ee_*s*eed*s*_) (ee_Δ_ ≈ ee_*s*eed*s*_ or ee_Δ_ > ee_*s*eed*s*_, respectively). Based on this concept, they proposed a chiral amplification constant, ee_Δ_/ee_*s*eed*s*_, and established an equation to correlate it with the mass of the added seeds (*m*_seeds_), making *m*_seeds_ an adjustable parameter to enhance ee_Δ_. The results predicted by this equation indicated that seeds with a mass reduced to only 1/50 of the original and only 60% ee_seeds_ still produced 90% ee_Δ_ and considerable symmetry-breaking. Moreover, this design strategy is also effective for other Cons, racemizing agents, and previously reported VR/TCID kinetic parameters, although it seems counterintuitive to realize high enantiopurity by using a small amount and low ee seeds. However, this study approximates ee_Δ_/ee_seeds_, which actually showed a downward trend, as a constant. Therefore, simply reducing *m*_seeds_ may not be necessarily beneficial to higher ee values. Also, the evolution of the crystal–solution interface and homochiral pre-nucleation clusters still remains ambiguous and needs further investigations.

**Fig. 20:**
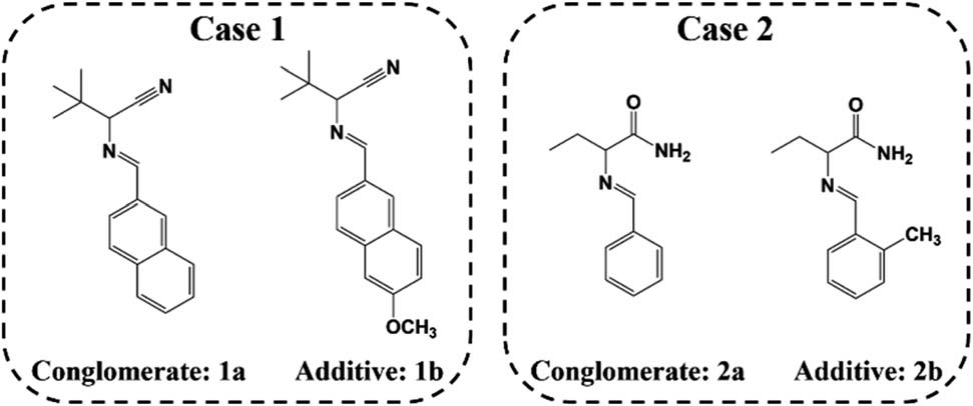
Chemical structures of the two families of compounds.^85^

Considering that chiral additives (Section 4.2.3) can inhibit the crystallization kinetics of specific enantiomers, the “rule of reversal” can also be used to entice directional chiral amplification. For instance, during the deracemization process of dl-amino acids observed under polarized light microscopy, switching the chirality of the additive, ethylenediammonium sulfate, induced a completely opposite color evolution pathway of the product crystals.^278^ This suggests that chiral additives, serving as heterogeneous impurities, can speed up TCID and VR and promote desired outcomes of mirror symmetry breaking.^273^ Additionally, chiral additives can occupy some sites within the crystal lattice and form a solid solution with a specific enantiomer. In response to this phenomenon, in 2020, Willem L. Noorduin and coworkers proposed an innovative resolution strategy to enrich the desired enantiomer in the solid phase. Specifically, the *S*-additive exclusively forms a solid solution with the *S*-enantiomer of the Con (Fig. 20). Although the solid solution, (*S*)-1a: (*S*)-1b, is thermodynamically less soluble than (*S*)-1a, its nucleation induction period is longer than (*R*)-1a. Therefore, (*R*)-1a crystals, which can be crystallized due to kinetic advantage, will continuously dissolve after reaching the peak of precipitation, while (*S*)-1a: (*S*)-1b will eventually become dominant in the solid phase under the control of thermodynamics. By comparison, the precipitation of the solid solution, (*S*)-2a:(*S*)-2b, is a completely thermodynamically favored process. However, its solubilizing effect on (*R*)-2a is nearly twice that of (*S*)-2a. Therefore, (*S*)-2a:(*S*)-2b and (*R*)-2a will be consistently enriched in the solid phase and liquid phase, respectively.^85^ This study revealed a potential VR/TCID itinerary when tailor-made additives can form solid solutions with enantiomers. The exploration of competition between thermodynamic-controlled “solid solution formation” and kinetics-determined “nucleation and growth inhibition” remains a challenge in achieving chiral amplification.

**Fig. 21:**
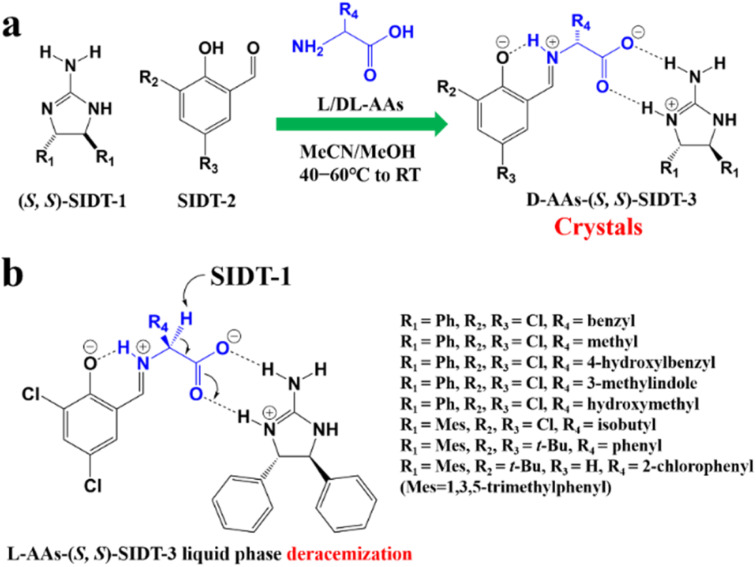
(a) Substrates for SIDT strategy of l to d conversion and deracemization. (b) Acid/base catalysis-based mechanism for l-AAs-(*S*, *S*)-SIDT-3 racemization (or dl-AAs deracemization).^40^

Generally, diastereomer-formation-related deracemization barely depends on the solubility difference and it can be predesigned, while crystallization-induced diastereomer transformation (CIDT) is an unknown process.^176^ For example, Flood *et al.* described a CIDT process using *S*/dl- and *S*/d-mandelamide diastereomers formed between D-/l-mandelic acid and (*S*)-1-phenylethylamine. The *S*/l epimer unexpectedly served as a stable crystal, while the *S*/d epimer served as a metastable gel phase because of the lack of strong π interactions within its predicted crystal structure. Therefore, the *S*/d epimer was converted to kinetically favored *S*/l crystals during CIDT with >92% ee and >70% yield.^279^ To subdivide diastereomer-related deracemization, new terminology, solubility-induced diastereomer transformation (SIDT), was pioneered to describe the “misnamed CIDT cases”, where the more soluble diastereomer was consecutively converted to the less soluble one by intermolecular interaction-triggered epimerization.^40^ Specifically, SIDT was applied to transform hard-to-racemize l-α-amino acids (AAs) to their counter-enantiomers with high profit margins. It can be observed that two resolving agents, SIDT-1 and SIDT-2, showed more close-knit binding toward d-AAs through one O–H⋯N and double N–H⋯O hydrogen bonds (Fig. 21a). By comparison, l-AAs-SIDT-3 underwent a fast racemization mechanism. AAs' α-proton could be deprotonated by neutral SIDT-1 (Fig. 21b), which may push the proton of N (guanidinium, SIDT-1) to O (−COO^−^, AAs) and allow the inversion of the chiral center. Consequently, stable d-AAs-SIDT-3 with lower solubility could be enriched in the solid phase, while l-AAs-SIDT-3 was racemized in the liquor phase, and nine dl/l-AAs (Fig. 21) could be deracemized to d-forms with considerable yields (70–91%) and de values (>94%). Although this theory has the potential to achieve deracemization through intermolecular interactions without the need for the derivatization of AAs, it seems that the explicit boundary between CIDT and SIDT merits further investigation.

Overall, crystallization-based deracemization ingeniously amplifies the initial asymmetry of the system by combining the racemization and crystallization kinetics of enantiomers. VR and TCID as economical methods exhibit desirable potential to deracemize all crystallizable Cons by coupling them with seeds (PC), chiral additives (reversed PC), liquid exchange processes, *etc.* Moreover, transforming Rac/Ss into racemizable Con *via* chemical derivatization and conciliating the incongruity between racemization and crystallization can further orient these methods to complex racemates. However, the complex interplay between chemical reactions and crystallization can lead to various deracemization mechanisms (CIDT, SOAT, SIDT, VR, TCID, *etc.*). Hence, it is necessary to incorporate racemization kinetics into well-established PBMs to develop novel models to direct total chiral amplification.

## 6. Porous materials method

Chiral porous materials (PMs), which possess excellent porosity and inherent chirality, strong intermolecular interactions, abundant chiral recognition sites and steric hindrance, are considered potential alternatives to ELLE and crystallization-based methods. Among them, MOF and COF materials have advantages due to their mature synthesis routes, high stability, tunable pore structures and functionalities, and abundant available building units. In comparison, other chiral PMs have advantages such as higher solution processability, milder and easier synthesis routes, and ease of regeneration. However, they face challenges of higher costs and lower productivity compared to crystallization/extraction techniques. Therefore, their application is currently limited to laboratory-scale separations, whereas the mass production of high-performance PMs is limited using this technique.

Homochiral MOFs and COFs are the most widely explored materials for chiral separation. Various types of building blocks have been designed and synthesized to modify the pore size and accommodate diverse model compounds.^280^ Given that they have been exhaustively introduced by many excellent reviews, here we offer a brief summary of important MOFs and COFs serving as CSPs or adsorbents reported over the last decade to avoid repetitive narratives (Table 4). It can be seen that cyclodextrin-/amino acid-based MOFs and COFs are trendsetting, and 2D MOFs with abundant chiral void spaces have become strong competitors to 3D materials. Notably, two crown ether-based PMs significantly expand the substrate scope and enhance the stability of CSPs in both HPLC and GC. Even after thousands of sample injections, the porous structures remain intact, and their performance remains comparable to commercially utilized columns.^281,282^ Hence, crown ethers are expected to produce novel CSPs with excellent resilience, enantioselectivity, efficiency and low crystal defects. However, the resolution outcomes vary drastically with materials. This is mainly attributed to the pore size effects of MOFs and COFs, as well as the differences in the uniformity and shape regularity of the prepared CSP particles.

**Table 4:** Some case studies of MOF- and COF-induced chiral resolution

Material	Chiral selector	Racemate	Enantioseparation performance	Ref.
**Chiral metal–organic frameworks**				
CD-MOF-1	γ-CD	1-Phenylethanol, pinenes and limonene	*α*_*R*-(+)-limonene_ = 1.72	283
			*α*_*S*-(−)-1-phenylethanol_ = 2.26	
TAMOF-1	(*S*)-3-(1*H*-Imidazole-5-yl)-2-(4*H*-1,2,4-triazol-4-yl)propanoic acid	Ibuprofen and thalidomide, 1-phenylethan-1-ol, benzoin, flavanone and *trans*-2,3diphenyloxirane	*α*_(*S*)-ibuprofen_ = 2	72
			*α*_(*R*)-thalidomide_ = 4	
(*R*)-CuMOF-1 (*R*)-CuMOF-2	(*R*)-3,3′-Bis(6-carboxy-2-naphthyl)-2,2′-dihydroxy-1,1′-binaphthyl	Methyl/ethyl/vinyl phenyl sulfoxide	*α* = 11.6–13.2	284
[DyNaL(H_2_O)_4_]·6H_2_O	1,1′-Biphenol derivative	Methyl mandelate, ethyl mandelate, isopropyl mandelate and benzyl mandelate	ee_*S*_ = 93.1%, 64.3%, 90.7% and 73.5%	285
[Mn_2_L^1^(DMF)_2_(H_2_O)_2_]·3DMF·2H_2_O	(*S*)-3,3′-Di-*tert*-butyl-5,5′-dibromo-6,6′-dimethylbiphenyl-2,2′-diol	2-Butylamine	*α* = 1.4	286
Cu_2_(Dcam)_2_(L)	(1*R*,3*S*)-(+)-Camphoric acid	Limonene	ee_*S*_ = 35%	287
2D-MOF sheets	Helical peptides	Tartrate and phenylalanine	ee > 99%	288
β-Cu_2_(LOBAla)(H_2_mbdpz)	*N*,*N*′-Oxalyl-bis(alanine)	1-Phenethyl alcohol and 1-phenyl-1-propanol	ee = 43.4% and 36.0%	289
D-His-ZIF-8	d-Histidine	dl-alanine and dl-glutamic acid	ee = 78.52% and 79.44%	290
D-His-ZIF-8@SiO_2_	d-Histidine	*trans*-stilbene oxide	*α* = 3.64	291
[Cd_9_((*R*)-PIA)_6_(TIB)_4_(H_2_O)_12_]·3H_2_O	Proline derivatives, (*S*)-/(*R*)-H3PIA	1-Phenylethanol and methyl lactate	ee_*R*_ = 21.2% and 34.8%	292
poly(l-DOPA) 2D thin film	l-DOPA	Naproxen	ee = 32%	293
[Zn(l(*d*)-Py-Thr)(H_2_O)(Cl)] 2D nanosheets	l-Threonine	Tris-oxalatochromium(iii) complex Δ-Cr(ox)_3_^3−^ and Λ-Cr(ox)_3_^3−^	ee_Λ-Cr(ox)3_^3−^ = 82%	294
			ee_Δ-Cr(ox)3_^3−^ = 78%	
Cu(Gly-l-His-Gly)	l-Histidine	Methamphetamine and ephedrine	ee = 30 ± 3% and 54 ± 2%	295
[(Zn_4_O)_2_(L)_6_(bpy)_3_]	(*R*)-/(*S*)-Phenylalanine methyl ester	Ibuprofen	ee > 99.9%	296
[Zr_6_(O)_4_(OH)_4_(L_*n*_)_6_] *n* = 1, 2, 3	L_1_: (*S*)-2-amino-1-propanol	Fmoc-l/d-Valine	ee_Val_ = 9.69%	297
	L_2_: l-valinol	Fmoc-l/d-Phenylalanine	ee_Phe_ = 5.26%	
	L_3_: (*S*)-2-phenylglycine	Fmoc-l/D-Tryptophan	ee_Try_ = 9.44%	
[Zr_6_O_4_(OH)_8_(H_2_O)_4_(L)_2_]	Crown ether	16 amino acid (derivatives) and 4 chiral drugs	Excellent for reversed-phase HPLC	281
[(HQA)(ZnCl_2_)(2.5H_2_O)]_*n*_	6-Methoxyl-(8*S*,9*R*)-cinchonan-9-ol-3-carboxylic acid	19 Racemic dansyl amino acids and 3 chiral drugs	Total baseline separation within 30 minutes	298

**Chiral covalent-organic frameworks**				
CTpPa-1	(+)-Diacetyl-l-tartaric anhydride	1-Phenylethanol, 1-phenyl-1-propanol, -limonene and methyl lactate	Fast separation within 5 minutes	299
CTpPa-2				299
CTpBD				299
(*R*, *R*)-CCOF-5	(*R*, *R*)-teraaldehyde	1-Phenyl-2-propanol	*α* = 1.19	300
(*R*, *R*)-CCOF-6	(*R*, *R*)-teraaldehyde	1-Phenyl-2-propanol, 1-phenyl-1-pentanol, 1-phenyl-1-propanol and 1-(4-bromophenyl)ethanol	*α* = 1.20–1.33	300
Silica-CCOF 17	Crown ether	Amino acids, esters, lactones, amines, alcohols, aldehydes, ketones, olefins, chiral drugs	Excellent for both GC and reversed-phase HPLC	282
Silica-CCOF 18				
Silica-COF-CD particles	β-CD	2-Phenylpropionic acid and 1-phenyl-1-propanol	Fast separation within 5 minutes	301

**Fig. 22:**
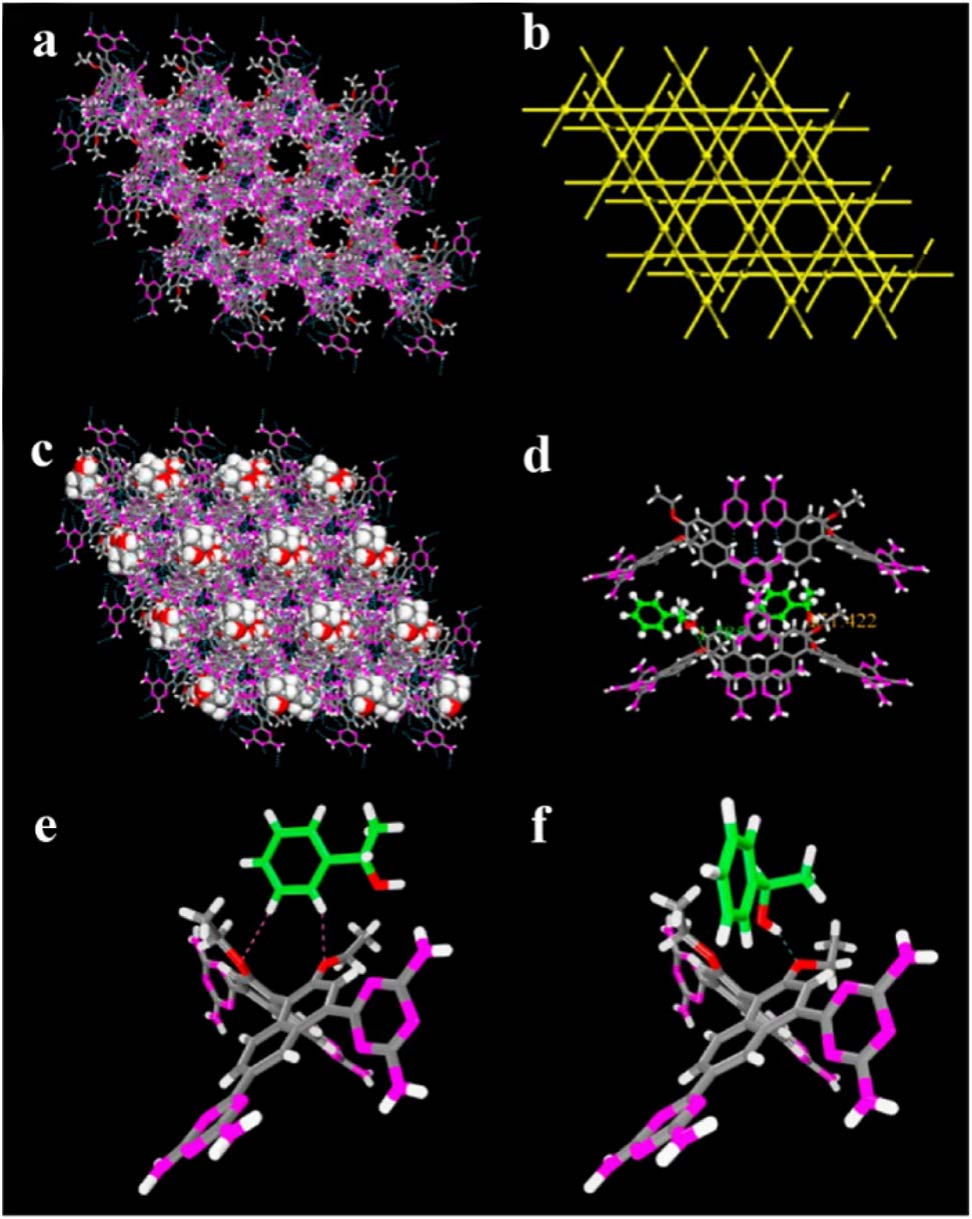
(a) X-ray crystal structure of HOF-2 featuring multiple H-bonds. (b) Uninodal 6-connected network topology. (c) X-ray crystal structure of HOF-2⊃(*R*)-1-PEA. (d) Chiral cavities of the framework for the specific recognition of (*R*)-1-PEA. Comparison of X-ray crystal structures of (e) HOF-2⊃(*S*)-1-PEA and (f) HOF-2⊃(*R*)-1-PEA (C, gray; H, white; N, pink; and O, red).^69^ Reproduced with permission from ref. 69. Copyright 2014, the American Chemical Society.

Besides MOFs and COFs, hydrogen-bonded organic frameworks (HOFs), which are fabricated mainly *via* the hydrogen bonding-induced self-assembly of building units, exhibit various merits, such as good solution processability, ease of purification, and easy recovery. However, the weaker nature of hydrogen bonds in comparison to chemical bonds may lead to limitations concerning porosity and stability sustainability.^302^ At present, chiral HOF-2, whose building block consists of hydrogen-bonded (*R*)-1,1′-bi-2-naphthol and 2,4-diaminotriazinyl, serves as the most prominent HOF simultaneously possessing strong hydrogen bonding, high porosity, structural integrity and thermal stability, although the solvent must be removed from pores its (Fig. 22). Each building block coordinates with six abutting ones through π–π stacking and hydrogen bonding to establish hexagonal chiral pockets (*ca.* 4.8 Å). Hence, it can enantioselectively “cocrystallize” with six secondary alcohols in their respective racemic solutions. In the most outstanding case, (*R*)-HOF-2 encapsulated (*R*)-1-phenylethanol (PEA) through O–H⋯O interactions, while only frail C–H⋯O interactions existed in (*R*)-HOF-2⊃(*S*)-PEA (Fig. 22). This allowed the formation of the stable (*R*)-HOF-2⊃(*R*)-PEA complex and subsequent liberation of (*R*)-PEA with 92% ee. In 2022, an HOF-2-coated capillary column further achieved the successful resolution of amino acids, organic acids and ethers during the GC process, with separation factors in the range of 1.02–1.25 and duration of 3 min.^303^ However, HOFs are rarely reported in this field due to their lower toughness, chemical stability and structure integrity after removal of the solvent. Therefore, the development of novel chiral building units is of great importance for the advancement of HOF-based enantioseparation.

Porous organic cages (POCs) are another type of material incorporating guest molecules by non-covalent interactions. Their merits of intrinsic cavities, good solution processability and higher solubility in organic solvents help to promote CSPs with fewer crystal defects.^280^ In 2020, a hydroxyl-functionalized homochiral POC exhibited decent selectivity toward 39 racemates as CSPs in a GC column.^304^ Also, Chen *et al.* used MD and DFT calculations to predict the separation performance of an (*R*, *R*)-1,2-cyclohexane diamine-based POC, CC3-*R*, for (*RS*)-PEA. They discovered the optimal ratio of CC3-*R* to PEA and the binding energy of CC3-*R*⊃(*R*)-1-PEA, which was 28.5 ± 4.0 kJ mol^−1^ higher than that of CC3-*R*⊃(*S*)-1-PEA.^305^ Therefore, computational chemistry provides a concise approach for screening and optimizing PM materials. Recently, three more [4 + 8]-type chiral POCs, imidazolium-based NC1-*R*, (1*R*,2*R*)-1,2-diaminocyclohexane-based NC4-*R*, and (1*R*,2*R*)-1,2-diphenyl-1,2-ethylenediamine-based POC (C_120_H_96_N_12_O_4_), formed novel CSPs for HPLC by chemically bonding with thiol-functionalized SiO_2_ gel. During the enantio-separation of numerous chiral drugs, the latter two showed good separation factors, fast resolution ability and good stability, which could be complementary to NC1-*R*, commercially used CHIRALPAK AD-H, and CHIRALCEL OD-H columns.^306–308^ Nevertheless, POCs are currently restricted by their building blocks. Thus, future research should develop diverse POC structures having different channel sizes and cavity volumes, and higher adaptability.

**Fig. 23:**
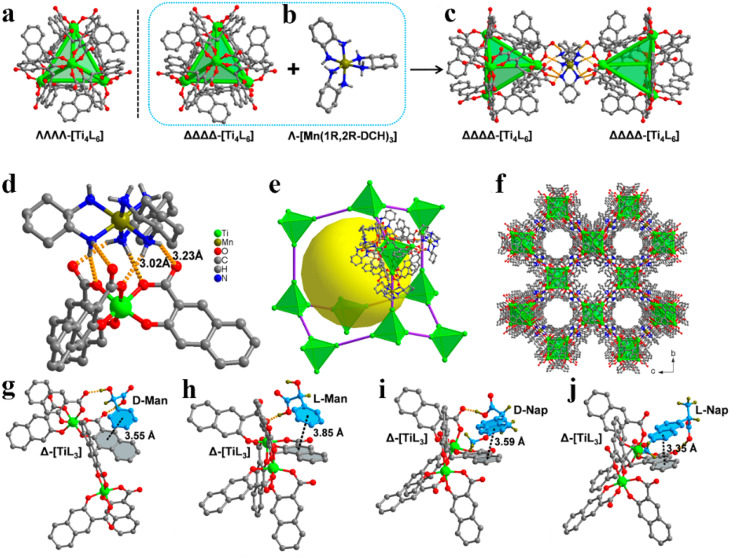
(a) Molecular structures of the ΔΔΔΔ-[Ti_4L6_] and ΛΛΛΛ-[Ti_4L6_] enantiomers. (b) Λ-[Mn(1*R*,2*R*-DCH)_3_] unit in PTC-108(Δ). (c) Two adjacent ΔΔΔΔ-[Ti_4L6_] cages linked by one Λ-[Mn(1*R*,2*R*-DCH)_3_] unit. (d) N–H⋯O H-bonds in PTC-108(Δ). (e and f) Diamondoid cage and 3D framework of PTC-108(Δ). (Ti, green; Mn, olive; O, red; N, blue; C, gray). (g–j) Illustration of the proposed recognition models of the PTC-108(Δ) cage towards enantiomers.^70^ Reproduced with permission from ref. 70. Copyright 2018, the American Chemical Society.

Furthermore, a novel type of chiral metal–organic cage (MOC) material, which shares the same building blocks as MOFs but exhibits limited structural diversity, molecular weight, and size, has been utilized for enantio-separation due to its good solution processability and the ability to bind chiral molecules both inside and outside the caves.^309^ For instance, Gu and coworkers innovatively synthesized an (*S*)-(MOF)(MOCs) film capable of the enantioselective absorption of (+)-methyl lactate by loading titanium-based homochiral MOCs on an achiral MOF.^310^ Also, He *et al.* ingeniously employed the enantiospecific cocrystal strategy (Fig. 5g) to simultaneously achieve the resolution of a racemic MOC, Ti_4_(L)_6_, and the synthesis of an enantiopure 3D MOC, PTC-108(Δ), for subsequent chiral separations (Fig. 23a–c).^70^ Specifically, the diamondoid structure of PTC-108(Δ) was fabricated through the self-assembly of chiral coformer Λ-[Mn(1*R*,2*R*-DCH)_3_] cations and anions ΔΔΔΔ-[Ti_4L6_] *via* N–H⋯O and C–H⋯O hydrogen bonding (Fig. 23d–f). Consequently, PTC-108(Δ) particles as an adsorbent still achieved enantioseparation ability for Rac mandelic acid (Man) and naproxen (Nap) despite their relatively small pore size of 10 Å. Further DFT calculations confirmed the enantioselectivity of PTC-108(Δ) by showing that it forms more O–H⋯O hydrogen bonds and stronger π–π interactions with d-Man and l-Nap, while the interactions with l-Man and d-Nap decrease to fewer and weaker ones (Fig. 23g–j). However, most chiral MOCs are synthesized *via* self-assembly in acetonitrile or aqueous solutions and cannot serve as stable solid materials, thereby reducing the available solvent systems for resolution.^311^ Meanwhile, compared to MOFs, MOCs often have limitations in the separation of large-sized enantiomers and are more prone to decompose under strict conditions of solvent pH, temperature, and concentration. Also, high concentrations of MOCs in solution can lead to aggregation, while low concentrations may not be sufficient for self-assembly, making the large-scale production of chiral MOCs a challenge. Consequently, it is important to promote MOC-based resolution by developing new solvent systems such as ionic liquids^312^ or encapsulating chiral MOCs into films/gels of existing MOFs.^310^

In addition, although the significant dependence of chiral porous organic polymers (POPs) on the aryl moiety reduces the available variety of materials, they remain potential candidates for CSPs due to their advantages of superior stability, processability and economic feasibility.^313–315^ For instance, Tan *et al.* synthesized a novel chiral POP, COP-1, by using Boc-3-(4-biphenylyl)-l-alanine as the building block and 4,4′-bis(chloromethyl)-1,1′-biphenyl as the crosslinker.^74^ The as-prepared COP-1 showed exceptional enantioselectivity toward PEA (*α* = 4.45), IBU (*α* = 10.18), and Nap enantiomers (*α* = 3.89) because of the stereospecific π–π interactions and H-bonds.^74^

Currently, the development of innovative chiral PMs has become the focal point of both chromatographic CSPs and membrane resolution because of their prominent host–guest interactions and steric hindrance. Chiral MOFs and COFs occupy the largest proportion in this field due to their tunable porosity, rigid structure and high stability. However, the most practical ones are still constructed from macrocycle-based (*e.g.*, cyclodextrin and crown ether) building blocks through *in situ* growth or post-synthesis modification. By comparison, amino acid-based ones usually show poor enantioselectivity. In addition, chiral POCs, MOCs, POPs, and HOFs are promising candidates with good processability and self-assembly potential. However, their relatively fixed pore sizes and limited types of building units limit their applications. Hence, it seems crucial to theoretically design PMs by DFT calculations and molecular dynamic simulations.

## 7. Membrane resolution method

**Fig. 24:**
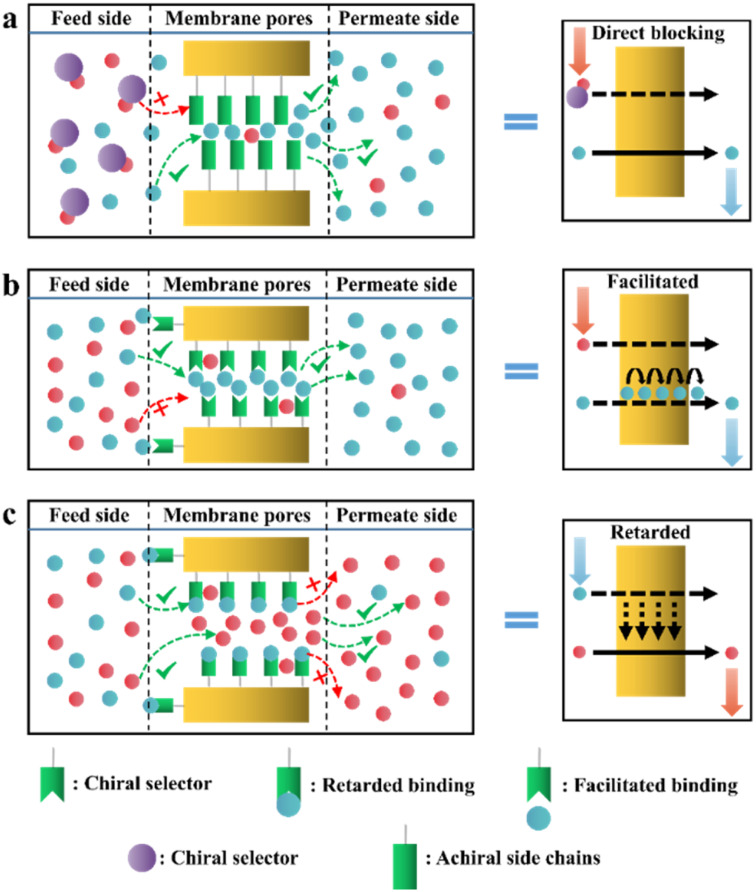
Enantioseparation based on (a) membrane-assisted process coupled with extraction or crystallization, (b) diffusion-selective membrane and (c) adsorption-selective membrane. Global results (right) and mechanism occurring in a diffusion cell (left).

Membrane processes, which have merits including low operating cost, high capacity, and ease of scalability, have become a promising candidate for chiral separation. One significant application involves membrane-enhanced ultrafiltration processes, where achiral materials are coupled with other resolution techniques, such as crystallization, extraction and deracemization. For example, enantiospecific complexes are directly blocked on the feed side and separated from the racemic solution based merely on the molecular size (Fig. 24a). Another application concerns enantioselective membranes, which include diffusion-selective membranes (DSMs) and sorption-selective membranes (SSMs).^316^ Specifically, DSMs can preferentially permeate the enantiomer firmly bound to the chiral recognition sites and enrich this enantiomer on the permeate side, thereby following the facilitated transport mechanism (Fig. 24b). However, a trade-off exists between permeability and permselectivity due to the slower non-selective diffusion of the competing enantiomer as a function of time.^317^ Alternatively, the significantly higher binding affinity of SSMs towards one enantiomer relative to the other results in the strong adsorption of this enantiomer on the membrane surface or within the channels, while its antipode diffuses to the permeate side, guided by the retarded transport mechanism (Fig. 24c). Although it can enhance both the selectivity and permeability, long-term operation may lead to more severe chiral contamination due to saturated chiral recognition sites. Consequently, for these membranes, it is crucial to conduct regular desorption. Nevertheless, two mechanisms coexist in practical membrane resolution processes given that facilitating the transport of one enantiomer also means retarding the transport of another. Hence, it is necessary to determine the dominant transport mechanism for realizing high enantioselectivity and permeate flux. Herein, the new cases of membrane resolution in recent years are elaborated primarily based on three types.

### 7.1 Diffusion-selective membranes

An enantiomer that has a strong binding affinity with DSMs can be transported to the permeate side *via* multiple “adsorption–desorption” cycles. Therefore, the pore size of the membrane, distribution of the chiral recognition sites, strength of the interactions, and type of driving force all affect the separation outcome.

**Fig. 25:**
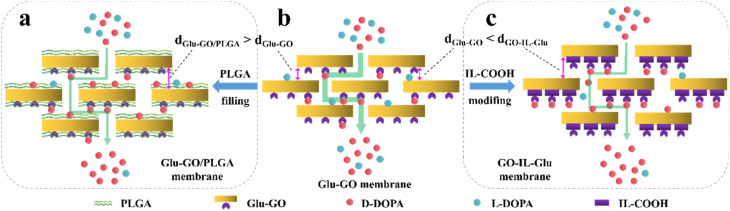
Stylized illustration of (a) Glu-GO/PLGA, (b) Glu-GO and (c) GO-IL-Glu composite membranes.^321,322^

In recent years, graphene oxide (GO) have emerged as a promising material for the fabrication of DSMs because of its abundant oxygen-containing functional groups, which provide ample sites for further derivatization.^318–320^ This facilitates the creation of tunable interlayer spacings and pore sizes, easy structural modification, and large surface area. For instance, Meng *et al.* successfully prepared a Glu-GO membrane with chiral channels and pores by chemically functionalizing GO membranes with a chiral selector, glutamic acid (Glu) (Fig. 25b).^318^ In concentration-driven permeation processes, the flux of d-DOPA exhibited a Langmuir-type curve, while that of l-DOPA experienced a simple linear curve with an increase in the feed concentration. To reduce the non-selective permeation of l-DOPA, the authors fabricated a membrane filled with polypeptide (PLGA) to achieve a more uniform distribution of chiral recognition sites between the Glu-GO layers (Fig. 25a).^321^ The as-synthesized Glu-GO/PLGA membrane resulted in a higher separation factor of 2.8 at a low feed concentration; however, the flux decreased to 75% of the previous Glu-GO membrane. Hence, the same group further increased the interlayer spacing and the number of chiral recognition sites by functionalizing the Glu-GO membrane with carboxyl-terminated ionic liquid (IL-COOH) (Fig. 25c). Consequently, both the separation factor and permeability of the GO-IL-Glu membrane were 2-times that of Glu-GO and 1.5-times that of Glu-GO/PLGA.^322^ Similarly, Liu *et al.* adjusted the interlayer spacing of a GO membrane to be approximately 8 Å *via* the introduction of l-cysteine (l-Cys). Concentration-driven permeation tests showed that the GO-Cys membrane exhibited significant resistance to molecules with a larger hydraulic radius of greater than 3.6 Å.^75^ Therefore, a high pressure difference can enhance the permeation rate and enantioselectivity during pressure-driven enantio-separations. However, it may induce the structure collapsing of the membrane, especially for chiral molecules with a size larger than the critical hydraulic radius of the membrane. In 2022, GO was also employed to enhance the specific surface and flow resistance of ethylenediamine-β-cyclodextrin (EDA-β-CD) enantioselective membranes. After using GO, the final ee values within a 24 h resolution process were improved from 56.99% to 100% for tryptophan and 4.55% to 71.47% for propranolol.^323^ In 2023, Wang for the first time utilized GO to enhance the resolution performance of a homochiral poly(2-oxazoline)-based (POx) membrane. Specifically, (*S*)-poly(2,4-dimethyl-2-oxazoline) (*S*-PdMeOx) was polymerized with a GO nanosheet on porous nylon layer-by-layer to fabricate a GO-backbone membrane. This modification enlarged the layer spacing from 0.75 to 0.94 nm and imparted the *S*-PdMeOx/GO membrane with abundant chiral recognition sites. Concentration-driven enantioseparation of (*RS*)-limonene in ethanol displayed 98.3% ee_*s*_ and 0.32 mmol m^−2^ h^−1^ flux despite the enhanced non-selective permeation of *R*-molecules as a function of concentration. Remarkably, the *S*-PdMeOx/GO membrane could maintain an intermediate performance of 54.6% ee_*s*_ and 0.18 mmol m^−2^ h^−1^ flux in the non-polar hexane solvent despite the partial collapse of the polymer network.^324^ As mentioned above, in future research, GO nanosheets can be utilized to prepare membranes with a uniform pore size, layer spacing and good chiral recognition site distribution. Characteristic reactions of advanced materials such as POx ring-opening polymerization can also be innovatively explored to construct novel chiral polymer networks.

**Fig. 26:**
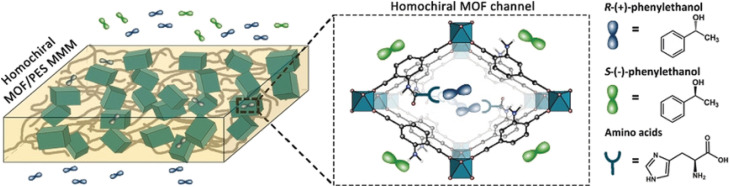
Schematic illustration of the selective transport of *R*-(+)- and *S*-(−)-1-phenylethanol through the MIL-53-NH-l-His channel.^325^ Reproduced with permission from ref. 325. Copyright 2019, John Wiley and Sons.

In addition, chiral mixed matrix membranes (MMMs) are novel materials obtained by blending chiral PMs with a polymer matrix. Specifically, the matrix imparts chiral PMs with improved morphology, desirable mechanical stability and scaling potential, while PMs provide abundant spatially uniformly distributed chiral recognition sites for the achiral membrane. Hence, MMMs possess chiral pore structures at both the membrane and PM scales, significantly enhancing the performance relative to individual chiral membranes or PM particles. For example, Lu *et al.* constructed MMMs by loading l-histidine (His) or l-glutamic acid (Glu)-functionalized MOF nanocrystals, MIL-53-NH-l-His or MIL-53-NH-l-Glu, on a polyethersulfone (PES) matrix, respectively (Fig. 26). Specifically, *R*-(+)-1-phenylethanol (*R*-PEA) could be selectively adsorbed by the MIL-53-NH-l-His/Glu nanocrystals than its antipode and preferentially permeated through MMMs with 100% ee_*R*_ in a concentration-driven diffusion cell, which significantly outperformed the purity achieved *via* simple adsorption using MOF particles (71% ee_*R*_).^325^ This is because the free volume of the MOF nanocrystals resembles that of the PES membrane, and thus loading MOFs would not block the channel of PES. Furthermore, developing PMs compatible with multiple amino acids is a cost-effective approach to improve the molecular flux and selectivity by adjusting the amino acid chiral selector. However, the permeation flux and ee value of *R*-PEA will inevitably decrease after reaching permeation equilibrium due to the competition of high concentration *S*-PEA for chiral recognition sites and the enhanced non-selective permeation over time. It should be noted that the PMs listed in Table 4 do not necessarily possess high enantioselectivity, which may arise from the insufficient contacts and mismatched pore sizes between enantiomers and chiral pores. However, embedding PMs in MMMs and imposing a driving force (concentration/pressure/potential difference) for enantioseparation can greatly enhance their performance.

**Fig. 27:**
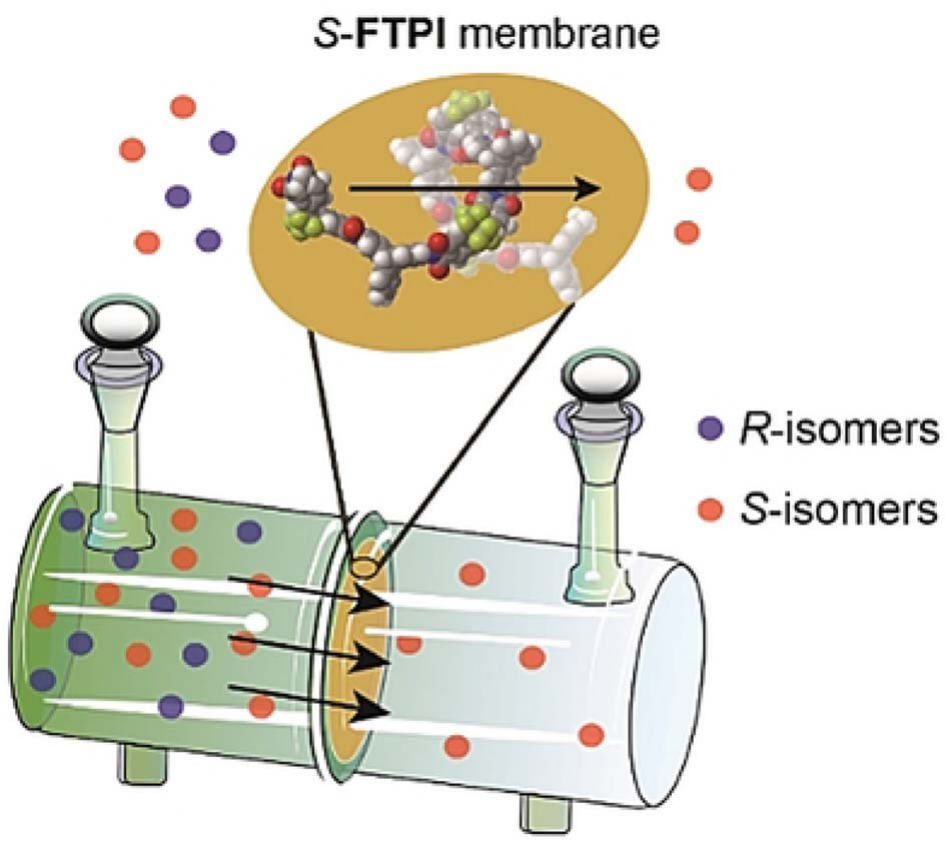
Schematic representation of triptycene-based chiral porous polyimides for enantioseparation.^326^ Reproduced with permission from ref. 326. Copyright 2021, John Wiley and Sons.

Additionally, triphenylethylene (derivatives) possesses inherent 3D rigid structures and high porosity, making it a promising candidate for synthesizing chiral membranes. In 2021, Zhang *et al.* synthesized an (*S*)-2,6-diaminotriptycene-based chiral porous polyimide (*S*-FITP) membrane. The adsorption of antipodes on the membrane was non-enantiospecific, thereby undergoing the facilitated transport mechanism during “adsorption–desorption” cycles. Depending on the good size-matching between the membrane pore and the molecular size, the *S*-FITP membranes could preferentially permeate *S*-enantiomers in a diffusion cell during a concentration-driven process, which led to ee values of 96.6% for (*RS*)-1,1′-binaphthyl-2,2′-diol, 11% for (*RS*)-2-naphthyl-1-ethanol and 7% for (*RS*)-mandelic acid (Fig. 27).^326^ Therefore, casting chiral polymers with high solution processability on achiral PMs is an important strategy to combine inherent chirality and rigid structures, which can mitigate the trade-off between permeability and selectivity.

Compared to chemical reactions, supramolecular self-assembly is a more ingenious and eco-friendly strategy for embedding PMs in membrane channels. In 2022, Huang *et al.* synthesized a chiral layer *c*CMS/AAO membrane only *via* O–H⋯O hydrogen bonding between the mesoporous chiral silica nanofibers (*c*CMS) and achiral porous anode alumina oxide (AAO). Under voltage-driven conditions, the membrane exhibited high separation factors in permeating l-amino acids from their racemate (7.52, 6.29, 2.53 and 1.75 for Arg, His, Asp and Glu, respectively).^327^ Notably, the abrupt changes in membrane pore size and ζ-potential during the permeation revealed the separation mechanism, as follows: over the separation time, the l-enantiomer anions undergo sequential processes of selective adsorption in the channel, facilitated transport driven by concentration and voltage, and desorption on the permeate side. Therefore, only when the isoelectric point of the amino acid is higher than the pH of the solution, the concentration and voltage differences synergistically promote the desorption of amino acid ions, rationalizing the somewhat lower enantioselectivity of the membrane towards acidic amino acids.

Although DSMs exhibit good enantioseparation ability, higher driving forces, such as concentration or pressure differences, can inadvertently exacerbate the non-enantioselective permeation of undesired enantiomers and lead to the collapse of the membrane.^75,321^ By comparison, SSMs have been proved to be more commercially attractive membranes.^328^

### 7.2 Sorption-selective membranes

Compared to DSMs, SSMs usually possess a larger pore size and undergo the retarded transport mechanism. Hence, these membrane processes are thermodynamically controlled, and their enantioselectivity and permeability can be simultaneously enhanced before reaching the saturation adsorption capacity.

One approach to achieve SSM-based resolution is simply functionalizing porous support materials with chiral molecules. For instance, in 2020, Gogoi *et al.* functionalized single-walled carbon nanotubes with d-tryptophan (Trp) and assembled them into a thin nanocomposite membrane to resolve dl-tyrosine (Tyr), enabling the permeation of l-Tyr with an ee value of 98.86%.^329^ In 2021, the same group anchored l-DOPA (or l-Trp) on double-walled carbon nanotubes to enhance the layer spacing and selectivity to develop tailor-made membranes for these two Racs. Consequently, 99% ee d-DOPA (or 98% ee d-Trp) could be permeated due to the presence of an additional hydrogen bonding/π–π interaction site between l-DOPA and membrane compared to d-DOPA.^330^ In the same year, 3D chiral conjugated microporous polymer (CCMP) SSMs were prepared by grafting a chiral selector on the surface of an SiO_2_ substrate. The as-prepared C_D_CMP-1/SiO_2_ membranes exhibited preferential permeation of l-enantiomers (94.1% ee for dl-Phe and 84.9% ee for dl-Trp), while C_D_CMP-1 powders possessed chiral preference for the d-enantiomers.^331^ In 2022, Qiu *et al.* functionalized cellulose fibres with β-CD-ionic liquid (β-CD-IL), rather than the traditional β-CD. Consequently, this enhanced membrane not only increased the binding energy for both enantiomers by 40% but also enlarged the binding affinity difference due to the improved π–σ interaction, π–π interaction, and hydrogen bonding in the β-CD-IL cavities.^332^ However, although these SSMs are relatively easy to fabricate, the porous environment only exists on the membrane scale and their channel size is difficult to adjust, which can result in relatively average selectivity and permeability. Therefore, a more advanced membrane resolution approach may be related to loading PMs such as MOFs and COFs on achiral porous support layers to fabricate MMMs.

**Fig. 28:**
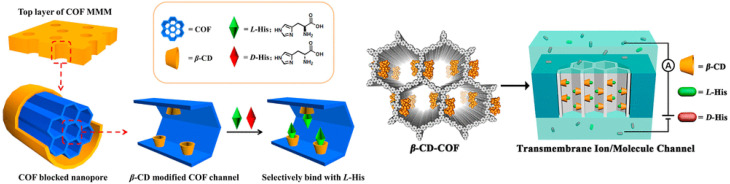
Schematic representation of the construction of β-CD-COF MMMs and subsequent voltage-driven enantioseparation.^333^ Reproduced with permission from ref. 333. Copyright 2019, the American Chemical Society.

Except for a few reports in Section 7.1, the majority of chiral MMMs can be affiliated to SSMs due to their high density of chiral recognition sites. For example, in 2021, a CD-MOF/PES MMM consisting of γ-CD-MOF and PES displayed preferential permeation for *R*-PEA, while the CD-MOF particles alone showed enantioselective adsorption toward *S*-PEA.^78^ Consequently, the 100% ee value for *R*-PEA could be maintained for about 24 h. Notably, the insufficient content of CD-MOF may lead to inadequate chiral recognition sites, while an excessive amount can cause agglomeration. Therefore, the loading amount of PMs and appropriate solvents are critical variables that determine the resolution outcome. In another example, β-CD-based chiral COF particles were used to fill the channels of an achiral membrane and fabricate MMMs capable of resolving amino acids (Fig. 28). In voltage-driven transmembrane transport, the separation factors of β-CD-COF MMM for dl-His, dl-Trp, and dl-Tyr reached 34.0, 2.0, and 1.5, respectively, at a pH value close to the isoelectric point of His, while significantly higher than that of Trp and Tyr. Hence, the low enantioselectivity for Tyr and Trp can be attributed to the electrostatic repulsion between negatively charged enantiomers and inner walls of MMMs. Interestingly, the separation performance of a physical mixture of β-CD-COF particles and polymer matrix is even superior to that of an MMM with an increased loading of β-CD in the inner walls of COFs.^333^ Hence, an excessive amount of chiral selectors can reduce the size compatibility between the PMs and the target molecules and lead to the loss of both enantioselectivity and permeation flux.

**Fig. 29:**
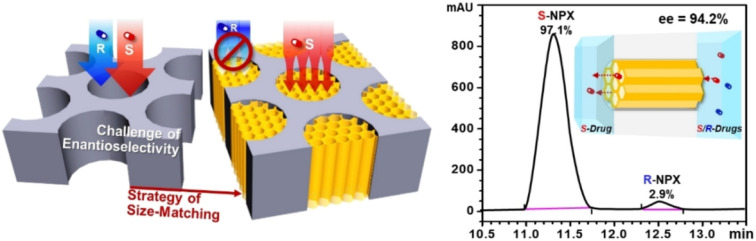
Schematic representation of chiral COF-packed nanochannel membrane to solve the challenge of enantioselectivity and HPLC chromatogram of separated naproxen through the l-Tyr-COF channel membrane.^76^ Reproduced with permission from ref. 76. Copyright 2022, John Wiley and Sons.

Besides MMMs, one approach to boost enantioselectivity is increasing the chiral selector density in local channels. In 2023, to produce membranes with good enantioselectivity, stiffness and strength, Milovanovic *et al.* innovatively utilized alkaline phosphatase to calcify a cross-linked (*R*/*S*)-*N*-(hydroxybutan-2-yl)amide hydrogel membrane in a buffer to establish an organic–inorganic double network.^334^ In this as-mineralized membrane, swelling in methanol could expand the pores occupied by polymers due to the good stretching ability of the inorganic calcium framework. Also, exposure to toluene could partially collapse the organic phase in the inorganic phase and *in situ* produce cavities. The high porosity and toughness exposed numerous chiral selector sites for enantioseparation in both polar and non-polar solvents. Consequently, this membrane realized considerable solute fluxes and enantioselectivity for (*RS*)-naproxen (in MeOH) and (*RS*)-BINOL (in toluene) during filtration. Hence, this strategy can be considered feasible to improve the membrane solvent resilience although the enantioselectivity may significantly decrease as a function of time because of the collapsed pores. Another novel utilization is developing tailor-made size-matching membranes toward target enantiomers. In 2022, l-tyrosine-modified COF (l-Tyr-COF) particles were *in situ* grown on a membrane from the channel surface to the center, which led to a polyethylene terephthalate nanochannel membrane with a decreased channel size from 400 to 2 nm (Fig. 29).^76^ This modification matched well with the molecular size of naproxen (Nap) enantiomers and increased the number of chiral recognition sites for l-Tyr. The l-Tyr-COF channels showed significant preference for (*S*)-Nap in both natural permeation and voltage-driven transport, which corresponded to a 16-times higher permeation flux of (*S*)-Nap (1.33 × 10^3^ μmol m^−2^ h^−1^) than its antipode. Furthermore, as the concentration of (*R*)-Nap increased, the current change induced by non-enantioselective permeation remained relatively low for l-Tyr-COF channels but increased correspondingly for l-Tyr channels. This means l-Tyr-COF channels boast enhanced enantioselectivity than the bare l-Tyr channel due to the compact packing of chiral selectors and sufficient recognition in the size-matched pores. According to Gaussian simulations, the former channel exhibited a 3-times higher Gibbs free energy difference and a doubled binding constant (for *R*/*S*-enantiomer complex) than the latter. Consequently, a separation factor 21.7-times that of the l-Tyr membrane and 94.2% ee_*S*_ could be achieved from racemic the Nap solution. Currently, these two strategies serve as cutting-edge techniques to reinvigorate previously reported low-enantioselective membranes.

**Fig. 30:**
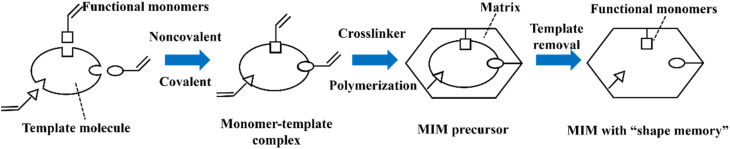
Schematic representation of the mechanism of MIM-induced chiral resolution.

In addition, significant research has focused on the study of chiral resolution by means of chiral molecularly imprinted membranes (MIMs), which possess much higher mechanical strength, stronger binding affinity and “shape memory”.^335^ Specifically, the target template (undesired enantiomer) and achiral functional monomers form a template-monomer host–guest complex through covalent or non-covalent interactions (Fig. 30).^79^ With the addition of the crosslinker, this complex can be immobilized on the polymer matrix to form an MIM precursor. Therefore, the removal of the template molecule leaves behind a cavity that memorizes the spatial structure, molecular property, and functional residue of the monomers, enabling chiral MIMs to enantiospecifically absorb the template enantiomer from the racemic solution. For example, Zhou *et al.* developed a green MIM only employing the pollutant-free substances d-tryptophan (Trp) (template), water (solvent), natural sodium alginate (functional polymer), CaCl_2_ (crosslinker), and PVDF (matrix). Noteworthy, the “shape memory” is based on the electrostatic repulsion force between the negatively charged d-Trp and –COO^−^ of sodium alginate. Therefore, in the pressure-driven permeation procedure, the pH value of the racemic feed solution should be higher than the isoelectric point of Trp to retain the chiral environment of the functional polymer. Under the optimal conditions, the purity of Trp on the permeate side reached 98% ee.^335^

In 2022, Mabrouk and coworkers reported the preparation of an innovative racemic MIM consisting of (*RS*)-ketoprofen (KET) (template), chitosan (functional monomer) and glutaraldehyde (crosslinker). Compared to (*S*)-KET-MIM, (*RS*)-KET-MIM exhibited exceptional cost-effectiveness (two-thirds lower than the cost of (*S*)-KET-MIM), but only one-third the enantioselectivity of (*S*)-KET-MIM (*α* = 2.31).^336^ Hence, MIMs can be alternatives to bypass the utilization of difficult-to-obtain or expensive single enantiomers. In the same year, a β-CD derivative having an incorporated l-Phe template in its cavities was grafted on the surface of a cellulose membrane *via* polymerization to undergo an electrodialysis process. The as-prepared l-Phe-MIM was placed between the acceptor and donor channel in an electrodialytic separation device. Under a 20 V voltage, the l-Phe enantiomer was enriched in the acceptor cell, while d-Phe was left in the donor side within 15 min (*α* = 2.95).^337^ Furthermore, an (*S*)-amlodipine (AD) MIM produced on the surface of a PVA membrane through a single-step graft/crosslinking procedure involved a transdermal transport process. In the vitro skin permeation cell, (*S*)-AD-MIM and rat skin were placed between the donor and acceptor cell in succession to simulate the percutaneous drug delivery process of (*RS*)-AD. Consequently, the cavities in MIM could stereoselectively release the *S*-enantiomer in the “body” with an enantioselectivity of 4.6 and a total amount of 1719.68 μg cm^−2^ (8 times higher than *R*-enantiomer) within 24 h.^338^ This means that MIMs possessing tailor-made channels may serve as plasters to pre-filtrate the undesired enantiomer before absorption by the skin.

According to the above-mentioned studies, it can be seen that the use of MMMs is a valuable strategy to improve the resolution performance and expand the substrate scope compared to normal chiral membranes. However, achieving complete enantioselectivity remains challenging for many chiral SSMs due to the unstable chiral recognition ability of PMs.^326^ Alternatively, MIMs exhibit superior adsorption stability and specificity, while their applicability is limited to the racemate of the template and are relatively lee cost-effective.^81^ Hence, there is still significant room for the future development of SSMs.

### 7.3 Membrane-assisted techniques

Unlike the above-mentioned membrane process, non-enantioselective membranes usually serve as enhancers that can be coupled with other resolution techniques. For instance, Wang *et al.* used l-isopentyl tartrate (l-IPT) and sulfobutylether-β-cyclodextrin (SBE-β-CD) as chiral selectors to form four complexes with ketolactone (KTZ) enantiomers in the ELLE process. Given that only l-IPT:(+)-KTZ and SBE-β-CD:(−)-KTZ serve as the stable diastereomer in the organic phase and aqueous phase, respectively, an achiral PVDF membrane that can permeate KTZ enantiomers but block complexes according to their molecular sizes (Fig. 24a) was placed between the two liquid phases. Consequently, the target (+)-KTZ enantiomer could be enriched in the organic phase with an ee higher than 90%.^339^ Therefore, the ELLE-membrane coupling process could reduce the contamination of chiral impurities in the target enantiomer phase, enhancing the selectivity of the ELLE process.

In addition, as discussed in Section 4.2.1, Racs with ee_0_ < ee_eu_ fail to be resolved through direct crystallization. Thus, to address this issue, in 2021, Horst and coworkers developed a membrane-assisted crystallization strategy for the resolution of three amino acid Racs (dl-Phe, dl-Ala and dl-Val).^191^ After the ultrafiltration of the starting solution containing dl-amino acid and BSA, d-amino acids could be permeated through the achiral membrane, while the enantiospecific complexes formed between l-amino acids and bovine serum albumin (BSA) could be blocked (Fig. 24a). When the ee value of the permeate side increased to exceed the ee_eu_, direct crystallization of the d-enantiomers could be achieved within ΔW_1_E_1_S, as shown in Fig. 7b. Therefore, this method is expected to serve as a malleable enrichment approach to apply direct crystallization to all Racs.

**Fig. 31:**
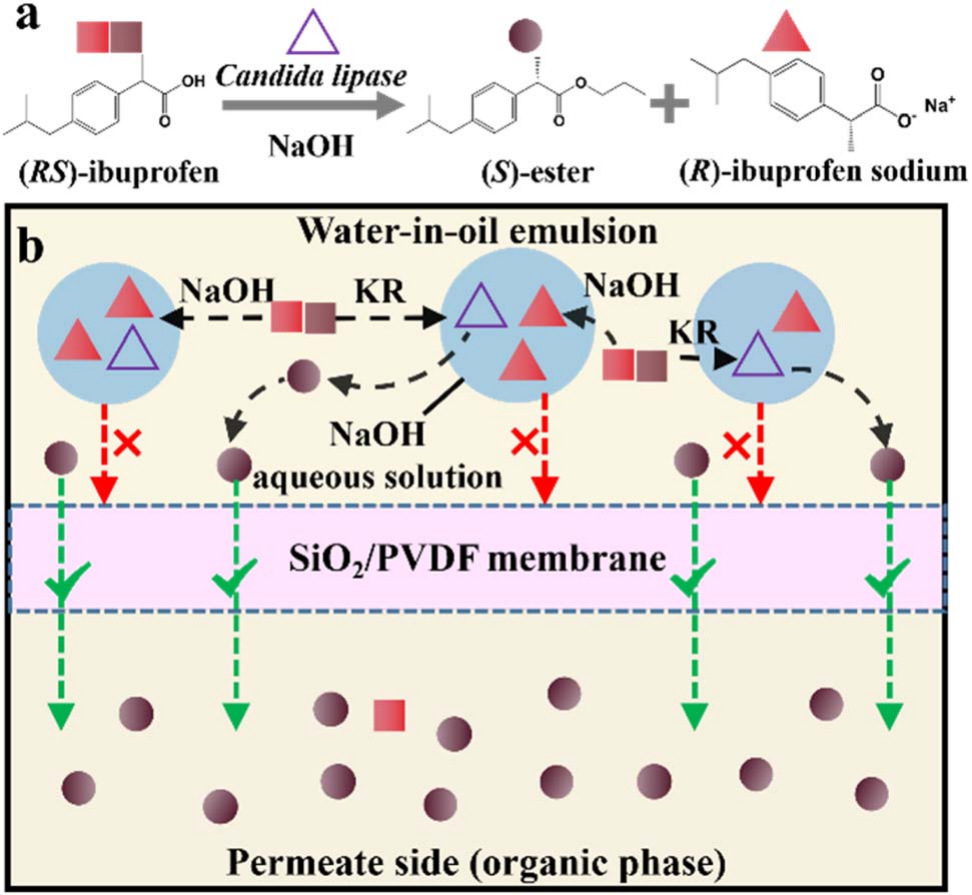
(a) Coupled strategy of membrane-enhanced enzymatic kinetic resolution by *Candida lipase* and (b) schematic representation of gravity-driven penetration of the reaction mixture through SiO_2_/PVDF membranes.^340^

Furthermore, in 2021, Maggay and coworkers proposed a membrane-enzymatic KR-coupled process for purifying enantiopure drugs from the water-in-oil emulsions.^340^ Under the synergistic effect of *Candida lipase* and gravity-driven filtration (Fig. 31a), the organic phase containing *S*-ibuprofen ester passed through the hydrophobic porous SiO_2_/PVDF membrane, while the non-esterified *R*-ibuprofen was neutralized by NaOH and enriched in the aqueous phase in the form of (*R*)-ibuprofen sodium (Fig. 31b). After tuning the porosity and pore size of the SiO_2_/PVDF membrane by adjusting the loading amount of SiO_2_, this coupled strategy could harvest nearly 100% optically pure *S*-enantiomer in the permeate side.

**Fig. 32:**
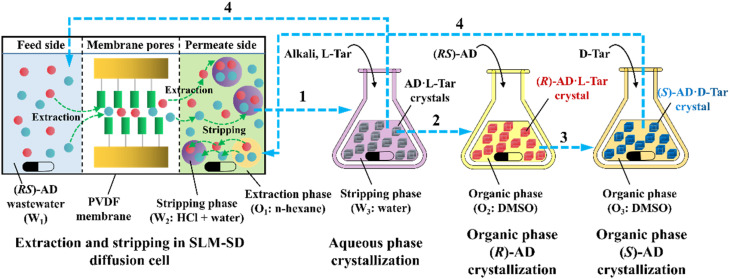
Schematic diagram of the integrated supported liquid membrane-aqueous/organic phase crystallization separation process (step 1: transfer of W_2_ from SLM-SD, step 2: transfer of aqueous phase crystal from W_3_, step 3: transfer of the mother liquor from O_2_, and step 4: mother liquor recycling routes of O_3_ → O_1_ and W_3_ → W_1_).^341^

Besides solid membranes, supported liquid membranes (SLMs), which are capable of coupling ELLE and stripping processes in a single unit, are also potential candidates due to their low operating costs and high industrial scalability.^129^ Recently, a novel SLM-ELLE-CCR coupling process was pioneered for recycling enantiopure amlodipine (AD) from its racemic wastewater (Fig. 32).^341^ The extraction and stripping process was integrated in a diffusion cell (SLM-SD) compartmented by an achiral PVDF membrane. The AD enantiomers could be extracted from W_1_ to O_1_ and stripped into W_2_, followed by the precipitation of (*RS*)-AD·1/2l-Tar crystals with the addition of l-Tar resolving agent (step 1). Using the two diastereomer crystals as the feed, (*R*)-AD·1/2l-Tar crystals could be harvested *via* cooling crystallization (step 2) and the (*S*)-AD-enriched mother liquor was mixed with d-Tar to produce (*S*)-AD·1/2d-Tar crystals (step 3). Ingeniously, the mother liquor recycling of the (*RS*)-AD-enriched solution (W_3_) and *R*-enriched DMSO (O_3_) (step 4) decreased the swelling issue of the PVDF membrane and increased the total yield. Consequently, crystal products with high purity and recovery could be obtained (ee_(*R*)-AD_ = 99.3% with 55.2% recovery rate, ee_(*S*)-AD_ = 99.8% with 53.9% recovery rate). Therefore, assisted processes integrating both merits of high ee of crystallization and high extraction yield are expected to streamline the setup and half the work with double the results.

Although non-enantioselective membranes fail to separate enantiomers alone, developing membrane-assisted processes have shown promise to surmount the bottleneck of other enantio-separation methods including ELLE, CCR, and KR.

## 8. Conclusions and outlook

Achieving an excellent separation performance can sometimes be difficult for individual methods because of their respective limitations and bottlenecks. Consequently, assistance from other methods is necessary to develop innovative coupling processes.

Specifically, the above-mentioned coupling techniques, such as ModiCon-VariCol,^99^ SFC-SMB,^102^ SMB-deracemization,^82,100^ ELLE-*in situ* crystallization,^52^ ELLE-membrane-crystallization,^51,147^ and ELLE-deracemization,^53^ demonstrate that ELLE and chromatography can be integrated with other enantioseparation methods and complement each other.

Cocrystal-based techniques, such as cocrystal-PE,^194,198^ CBR-deracemization,^239^ and CBR-PC,^243–245^ represent promising candidates for exploring enantioseparation mechanisms, expanding the substrate scope and enabling the efficient “reciprocal resolution” of complicated enantiomers. Hence, it will be a trendsetting topic to explore the mechanism of enantiospecific cocrystal formation using crystal structures and DFT calculations including the binding energy, crystal packing mode, and solvation free energy.^233^ Besides, coupling crystallization with ultrasound waves or magnetic fields may also raise unexpected discoveries in conglomerate preparation,^89,158^ enantioselectivity enhancing^216^ and process development.^190^

Meanwhile, the successful deracemization of complex racemates besides Cons through crystallization-based methods can also be attributed to the coupling strategy, as exemplified by chemical derivatization-VR-PC,^262^ chemical derivatization-TCID-PC,^91^ CBR-CIDT process with liquid-phase coupling,^239^ TCID-PC process with suspension exchange^86^ and reverse PC-VR-TCID.^85,273^ Consequently, it is indispensable to design novel coupled multi-vessel setups to overcome the trade-off between crystallization and racemization.

In addition, chiral membranes, which serve as coupled materials of a membrane matrix and chiral PMs, such as GO/MOF/COF, which incorporates chiral selectors, are promising candidates for enantioseparation. However, minimizing the trade-off between enantioselectivity and permeability seems the first concern. Consequently, it is necessary to increase the density of chiral recognition sites in the local channels by designing MMMs, size-matching membranes, and MIMs, imparting membranes with desirable mechanical stability, uniformly distributed chiral selectors, and scaling potential. Alternatively, achiral membranes can be coupled with direct crystallization, ELLE, CCR, and KR processes to overcome their respective limitations.^191,340,341^ The above-mentioned findings highlight the core concept that coupling strategies significantly benefit the six chiral separation methods. Nevertheless, the membrane regeneration and trade-off issues are inextricable shortcomings for membrane resolution. Hence, the construction of membranes with high toughness and stiffness, capable of *in situ* cleaning, seems to be a potential method.^342^

In conclusion, a comprehensive summary of the research progress and potential trends regarding six critical resolution methods was provided in this review. More importantly, the notable performance improvements achieved through the state-of-the-art “coupling” strategy were highlighted, which ingeniously injects new vitality into the field of enantioseparation.

## Abbreviations

PsC: Preparative-scale chromatography
HPLC: High-performance liquid chromatography
GC: Gas chromatography
LC: Liquid chromatography
SFC: Supercritical fluid chromatography
SMB: Simulated moving bed
CCC: Countercurrent chromatography
HSCCC: High-speed countercurrent chromatography
CSP: Chiral stationary phase
ELLE: Enantioselective liquid–liquid extraction
CD: Cyclodextrin
DES: Deep eutectic systems
HP-β-CD: Hydroxypropyl-β-cyclodextrin
Rac: Racemic compound
Con: Conglomerate
Ss: Solid solution
SHG: Secondary harmonic generation
PC: Preferential crystallization
CPC-D: Coupled preferential crystallization-dissolution
PE: Preferential enrichment
CCF: Chiral cocrystal coformer
Mal: Malic acid
PZQ: Praziquantel
Phe: Phenylalanine
Lys: Lysine
Val: Valine
Asp: Asparagine
Thr: Threonine
His: Histidine
Gly: Glycine
Trp: Tryptophan
PEA: Phenethyl alcohol or phenylethanol
AD: Amlodipine
CCR: Classical chemical resolution
CBR: Cocrystallization-based resolution
DR: Dutch resolution
D-DBTA: (+)-Dibenzoyl-d-tartaric acid
l-DBTA: (−)-Dibenzoyl-l-tartaric acid
d-DTTA: (+)-Di-*p*-toluoyl-d-tartaric
l-DTTA: (−)-Di-*p*-toluoyl-l-tartaric
d-DMTA: (+)-Di-*p*-anisoyl-d-tartaric acid
l-DMTA: (−)-Di-*p*-anisoyl-l-tartaric acid
PBMs: Population balance models
KR: Kinetic resolution
DKR: Dynamic kinetic resolution
CIDT: Crystallization-induced diastereomer transformation
VR: Viedma ripening
TCID: Temperature cycling-induced deracemization
SOAT: Second-order asymmetric transformation
CS: Chiral selector
Glu: Glutamic acid
Tyr: Tyrosine
MOFs: Metal–organic frameworks
COFs: Covalent organic framework
HOFs: Hydrogen-bonded organic frameworks
POPs: Porous organic polymers
POCs: Porous organic cages
MOCs: Metal–organic cages
Nap: Naproxen
ee: Enantiomeric excess
de: Diastereomeric excess
PXRD: Powder X-ray diffraction
SCXRD: Single Crystal X-ray Diffractometers
FT-IR: Fourier-transform infrared spectroscopy
Raman: Raman spectra
DSC: Differential scanning calorimetry
DFT: Density functional theory
MMMs: Mixed matrix membranes
MIMs: Molecular imprinted membranes
DSM: Diffusion-selective membranes
SSM: Sorption-selective membranes

## Author contributions

Jingchen Sui: conceptualization, methodology, visualization, writing – original draft. Na Wang: funding acquisition, supervision, writing – review & editing. Jingkang Wang: funding acquisition. Ting Wang: supervision. Xin Huang: supervision. Lina Zhou: funding acquisition. Hongxun Hao: funding acquisition, supervision, writing – review & editing.

## Conflicts of interest

The authors declare no competing interest.

## Supplementary Material
